# Apolipoprotein E imbalance in the cerebrospinal fluid of Alzheimer’s disease patients

**DOI:** 10.1186/s13195-022-01108-2

**Published:** 2022-11-02

**Authors:** Matthew Paul Lennol, Irene Sánchez-Domínguez, Inmaculada Cuchillo-Ibañez, Elena Camporesi, Gunnar Brinkmalm, Daniel Alcolea, Juan Fortea, Alberto Lleó, Guadalupe Soria, Fernando Aguado, Henrik Zetterberg, Kaj Blennow, Javier Sáez-Valero

**Affiliations:** 1 Instituto de Neurociencias de Alicante, Universidad Miguel Hernández-CSIC, Av. Ramón y Cajal s/n, E-03550 Sant Joan d’Alacant, Spain; 2 Centro de Investigación Biomédica en Red sobre Enfermedades Neurodegenerativas (CIBERNED), Sant Joan d’Alacant, Spain; 3 Department of Cell Biology, Physiology and Immunology, Faculty of Biology, University of Barcelona, Barcelona, Spain; 4 Institute of Neurosciences, University of Barcelona, Barcelona, Spain; 5 Instituto de Investigación Sanitaria y Biomédica de Alicante (ISABIAL), Alicante, Spain; 6 Institute of Neuroscience and Physiology, Department of Psychiatry and Neurochemistry, the Sahlgrenska Academy at the University of Gothenburg, Mölndal, Sweden; 7 Sant Pau Memory Unit, Neurology Department, Hospital de la Santa Creu i Sant Pau, Biomedical Research Institute Sant Pau, Universitat Autònoma de Barcelona, Barcelona, Spain; 8 Barcelona Down Medical Center, Fundació Catalana Síndrome de Down, Barcelona, Spain; 9 Laboratory of Surgical Neuroanatomy, School of Medicine and Health Sciences, University of Barcelona, Barcelona, Spain; 10 Clinical Neurochemistry Laboratory, Sahlgrenska University Hospital, Mölndal, Sweden; 11 Department of Neurodegenerative Disease, Institute of Neurology, University College London, London, UK; 12 UK Dementia Research Institute at UCL, London, UK; 13 Hong Kong Center for Neurodegenerative Diseases, Hong Kong, China

**Keywords:** Alzheimer’s disease, apoE, Biomarker, Aberrant complexes, Cerebrospinal fluid, Glycoform imbalance

## Abstract

**Objective:**

The purpose of this study was to examine the levels of cerebrospinal fluid (CSF) apolipoprotein E (apoE) species in Alzheimer’s disease (AD) patients.

**Methods:**

We analyzed two CSF cohorts of AD and control individuals expressing different *APOE* genotypes. Moreover, CSF samples from the TgF344-AD rat model were included. Samples were run in native- and SDS-PAGE under reducing or non-reducing conditions (with or without β-mercaptoethanol). Immunoprecipitation combined with mass spectrometry or western blotting analyses served to assess the identity of apoE complexes.

**Results:**

In TgF344-AD rats expressing a unique apoE variant resembling human apoE4, a ~35-kDa apoE monomer was identified, increasing at 16.5 months compared with wild-types. In humans, apoE isoforms form disulfide-linked dimers in CSF, except apoE4, which lacks a cysteine residue. Thus, controls showed a decrease in the apoE dimer/monomer quotient in the *APOE* ε3/ε4 group compared with ε3/ε3 by native electrophoresis. A major contribution of dimers was found in *APOE* ε3/ε4 AD cases, and, unexpectedly, dimers were also found in ε4/ε4 AD cases. Under reducing conditions, two apoE monomeric glycoforms at 36 kDa and at 34 kDa were found in all human samples. In AD patients, the amount of the 34-kDa species increased, while the 36-kDa/34-kDa quotient was lower compared with controls. Interestingly, under reducing conditions, a ~100-kDa apoE complex, the identity of which was confirmed by mass spectrometry, also appeared in human AD individuals across all *APOE* genotypes, suggesting the occurrence of aberrantly resistant apoE aggregates. A second independent cohort of CSF samples validated these results.

**Conclusion:**

These results indicate that despite the increase in total apoE content the apoE protein is altered in AD CSF, suggesting that function may be compromised.

**Supplementary Information:**

The online version contains supplementary material available at 10.1186/s13195-022-01108-2.

## Background

An important breakthrough in our understanding of Alzheimer’s disease (AD) was the identification of the apolipoprotein E *APOE-*ɛ4 allele as a risk factor [1]. Apolipoprotein E (apoE) protein is a component of lipoprotein particles in the plasma, as well as in the cerebrospinal fluid (CSF) [2]. ApoE regulates important signaling pathways by interacting with receptors and is present as sialylated glycoforms [3]. Human apoE lacks the consensus sequence necessary for N-linked glycosylation; thus, O-linked carbohydrates probably account for glycosylation [4]. The impact of apoE glycosylation remains unclear, but evidence indicates that glycosylation acts as an important post-translational mechanism for fine-tuning apoE interaction with receptors and proteins [5].

In humans, three versions of the *APOE* gene exist, ε2 (apoE2), ε3 (apoE3), and ε4 (apoE4) alleles, while other mammals only have one version of the *APOE* gene, resembling ancestral apoE4 [6]. *APOE*-ε3 is the most common allele (~75%), followed by ε4 (15–20%) and ε2 (4–8%) [7]. Compared to the most common *APOE* ε3/ε3 genotype, each additional copy of the *APOE*-ε4 allele is associated with a higher risk of AD and a younger mean age of dementia onset. Thus, in individuals with one copy of the *APOE-*ε4 allele, the risk of AD increases 2–3 times and 8–12 times in individuals with two copies [8]. Experimental evidence shows the deleterious effect of the apoE4 variant for AD, while the lack of apoE4 appears to be protective [9]. In contrast, the presence of one or two copies of the *APOE*-ε2 allele is associated with a lower risk of AD and an older mean age of dementia onset [10]; therefore, it has been hypothesized that the apoE2 protein could be protective against AD [11]. Indeed, *APOE*-ε2 homozygotes present an exceptionally low likelihood of developing AD [12]. The reported effects of different *APOE* genotypes on AD risk vary widely with demographic factors such as gender and ethnicity [7]. Moreover, the percentage of *APOE* genotypes in cognitively unimpaired people with neuropathological or biomarker evidence of preclinical AD, or the percentage of people who meet the criteria for mild cognitive impairment with or without biomarker evidence of AD, is not well established (discussed in [12]). Anyhow, despite the 2–3-fold increase in AD prevalence in *APOE-*ε4 subjects compared to the general population, most of the individuals with AD are *APOE*-ε3 carriers [13].

Nonetheless, given the important physiological functions of apoE, a malfunctioning of the apoE protein may also contribute to AD pathology in ε4 non-carriers [14]. The differences in the structure of apoE isoforms influence their ability to bind lipids, receptors, and amyloid-β (Aβ), which aggregates in plaques within the brain of AD patients [14].

Interestingly, apoE forms disulfide-linked homodimers and heterodimers with the apoA-II apolipoprotein involving the cysteine (Cys) at position 112 [7, 14]. Indeed, these apoE homodimers linked by disulfide bonds could be the native form able to bind to receptors [15]. The three human apoE isoforms differ in the presence of Cys/arginine (Arg) at positions 112 and 158 within the receptor binding domain, as apoE4 lacks Cys residues at both these positions [4]. The amino acid substitution of Cys-112 by Arg in apoE4 explains the lower number of disulfide-linked dimers in the CSF of *APOE* ε3/ε4 subjects compared with *APOE* ε3/3 subjects, and their absence in *APOE* ε4/ε4 subjects [16, 17], but may also explain the reduced ability of apoE4 to mediate some of its biological roles, compared with apoE2 or apoE3 [18].

The mature apoE protein has 299 amino acids and a molecular mass of ~35 kDa. However, previous studies performed in the brain [19] and CSF [17] reported a ~100-kDa apoE band in non-reducing conditions, as opposed to the predicted ~70 kDa, which was referred to as an apoE homodimer.

Previous studies that considered total CSF apoE levels failed to demonstrate consistent changes when the *APOE* genotype was included as a covariate in the models [20–22]. However, other studies associated high CSF apoE concentrations with an increased risk of impaired cognitive progression in non-apoE4 carriers [23].

Anyhow, in order to consider the estimation of apoE levels in CSF as a read-out of AD occurrence or progression, in addition to the *APOE* genotype, the studies should also consider changes in the protein conformation/structure that can compromise the biological function of the apoE protein. In this study, we aimed to characterize the occurrence of different apoE species in AD CSF from individuals with different *APOE* genotypes, while considering changes in the balance of apoE glycoforms and the occurrence of aberrant apoE dimers that could indicate a compromise of apoE function in the brain.

## Materials and methods

### Patients

CSF samples from individuals with known *APOE* genotypes were obtained from two independent cohorts. The CSF samples from both cohorts used for this study were de-identified aliquots from clinical routine analyses, following procedures approved by the Ethics Committees at the University of Gothenburg and the Hospital Sant Pau, respectively. Additionally, this study was approved by the ethics committee at the Miguel Hernandez University, and was carried out in accordance with the Helsinki Declaration regarding research on humans.

The CSF samples were obtained by lumbar puncture and centrifuged (2000×g, 10 min) and then immediately aliquoted and stored in ultrafreezers and kept at −80°C until analysis. The time between CSF acquisition and storage was less than 4 h in all cases. The handling of the samples was performed following recommended operating procedures [24]. Freeze-thaw cycles were avoided and new aliquots were used for each independent analysis.

The first cohort was from the longitudinal geriatric population study in Piteå, Sweden [25], the Piteå Dementia Project. The diagnostic evaluation included a clinical examination (detailed medical history and somatic, neuropsychiatric, and neurological status), a neuropsychological test battery, routine blood and CSF tests, and a CT scan to exclude secondary dementias [26]. All clinical diagnoses and evaluations were made without knowledge of the results of the biochemical analyses and vice versa. The cohort consisted of 45 patients with AD (fourteen men and thirty-one women, mean age 77±1 years) and was selected based on the *APOE*-ε4 status, so that fifteen each had *APOE* ε3/ε3, *APOE* ε3/ε4, or *APOE* ε4/ε4. In addition, fourteen non-AD controls [seven men and seven women, mean age (67 ± 3 years); *APOE* ε3/ε3: 9, *APOE* ε3/ε4: 5] were included. *APOE* genotype was determined by the solid-phase mini-sequencing method as previously described [27]. For this study, patients who were designated as AD or controls also had typical core CSF biomarker levels [Aβ42 and total tau (T-tau)] using cut-offs that are >90% specific for AD [28], but except for CSF Aβ42 and T-tau, all biochemical analyses were made without knowledge of the clinical data. The ethics committees in Umeå University and University of Gothenburg approved the study.

The second cohort was obtained from the Sant Pau Initiative on Neurodegeneration (SPIN cohort) [29] from Hospital Sant Pau (Barcelona, Spain). We included samples from 29 AD patients (thirteen men and sixteen women, mean age 73±1 years; *APOE*: 10 ε3/ε3, 10 ε3/ε4, 9 ε4/ε4) and ten controls (seven men and three women, mean age 69±2 years; *APOE*: 5 ε3/ε3, 5 ε3/ε4). Typically, these are patients who present cognitive complaints and are referred to the specialized memory unit from their primary care physician. All patients undergo a full neuropsychological evaluation that demonstrates objective cognitive impairment. Patients were included in the cohort when they presented supportive biomarkers of the AD pathophysiological process. Cognitively normal participants were volunteers without cognitive complaints and normal neuropsychological evaluation. More details about inclusion/exclusion criteria and neuropsychological tests in this cohort are detailed elsewhere [29].

In this cohort, the *APOE* genotype was determined by direct DNA sequencing and visual analysis of the resulting electropherogram performed to identify the two coding polymorphisms that encode the three possible apoE variants [29].

Each center applied their own internally validated cut-offs, according to their preanalytical and analytical particularities. More details about the cut-offs applied are indicated below. Samples were retrospectively selected from large cohorts to balance age, sex, and *APOE* status. Most of the selected cases (92%, 43 of 45 from Gothenburg and 25 of 29 from Barcelona) were categorized A+T+ according to [30]; thus, subgrouping by the AT(N) system for analysis was impractical. For full details about the collections, see Table 1.

**Table 1:** Demographic and biomarker information from the CSF samples obtained from the Gothenburg (Sweden) and Barcelona (Spain) cohorts

**Cohort: Gothenburg (Sweden)**							
	**Control**			**Alzheimer’s disease**			
***APOE***	**ε3/ε3**	**ε3/ε4**	**All**	**ε3/ε3**	**ε3/ε4**	**ε4/ε4**	**All**
***N***	9	5	14	15	15	15	45
**Age (years)**	69±2	62±5	67±3	79±2	78±1	73±1	77±1*
**Age (range)**	60–81	44–75	44–81	62–88	69–84	63–83	62–88
**Female/male**	5/4	2/3	7/7	11/4	11/4	9/6	31/14
**CSF Aβ42 (pg/mL)**	845±96	746±121	804±74	470±13*	480±8*	419±21	457±10*
**CSF tau (pg/mL)**	317±53	303±34	312±35	816±88*	917±112*	731±53	840±52*
**Cohort: Barcelona (Spain)**							
	**Control**			**Alzheimer’s disease**			
***APOE***	**ε3/ε3**	**ε3/ε4**	**All**	**ε3/ε3**	**ε3/ε4**	**ε4/ε4**	**All**
***N***	5	5	10	10	10	9	29
**Age (years)**	71±2	67±52	69±2	75±2	73±2	72±2	73±1*
**Age (range)**	66–76	60–72	60–76	64–84	64–83	61–85	61–85
**Female/male**	1/4	2/3	3/7	7/3	2/8	7/2	16/13
**CSF Aβ42 (pg/mL)**	1139±248	1010±116	1075±131	607±60*	543±37*	493±62	549±31*
**CSF tau (pg/mL)**	295±49	261±23	278±26	778±94*	624±62*	908±81	765±50*

Values are represented as mean ± SEM. *Significantly different (*T-*test, *p*< 0.05) from the control group with the same *APOE* genotype or regardless of the genotype (“All” columns)

### Determination of AD core biomarkers by ELISA and definition of cut-offs

In the cohort from Gothenburg, the levels of the AD core biomarkers T-tau, P-tau, and Aβ42 were measured in the CSF using INNOTEST ELISAs (Fujirebio-Europe, Gent, Belgium). Patients were designated as AD or controls according to CSF biomarker levels using cut-offs that are >90% specific for AD: Aβ42 <550 pg/mL and total tau (T-tau) >400 pg/mL [20].

For the cohort from Barcelona, cut-offs for AD biomarkers measured in the Lumipulse automated platform (Fujirebio-Europe) were T-tau > 400 pg/mL, P-tau > 63 pg/mL, and 0.062 for the Aβ42/Aβ40 ratio [29].

All samples were analyzed as part of a clinical routine by board-certified laboratory technicians following strict procedures for batch-bridging, analyses, and quality control of individual ELISA plates.

### Transgenic rat CSF

The experiments were carried out using a cohort of 107 rats (53 males and 54 females), including transgenic TgF344-AD rats (*n* = 52) expressing mutant human APP (APPsw) and presenilin-1 (PS1ΔE9) genes [31] and wild-type Fischer rats (*n* = 55). Rats were bred in the animal research facilities at the University of Barcelona. Animals were provided with food and water ad libitum and maintained in a temperature-controlled environment in a 12/12-h light-dark cycle. CSF samples (50–100 μL) were collected from ketamine/xylazine-anesthetized animals by cisternal puncture with a glass capillary in the suboccipital region through the atlanto-occipital membrane, with a single incision into the subarachnoid space [32]. CSF aliquots from different time points [4 months: 16 wild-type (8 male, 8 female) and 16 TgF344-AD animals (8 male, 8 female); 10.5 months: 17 wild-type (8 male, 9 female) and 16 TgF344-AD animals (8 male, 8 female); 16.5 months: 22 wild-type (12 male, 10 female) and 20 TgF344-AD animals (9 male, 11 female)] were analyzed. This study was part of a large project assessing various different proteins that included brain analysis at each stage; thus, it was not possible to perform longitudinal measurements in the same animal (repeat sampling) to reduce the number of animals. Animal work was performed in accordance with the local legislation, with the approval of the Experimental Animal Ethical Committee of the University of Barcelona, and in compliance with European legislation.

### Western blotting

Samples of human or rat CSF (10 μL) were denatured at 98°C for 5 min and resolved by sodium dodecyl sulfate-polyacrylamide gel electrophoresis (SDS-PAGE) under reducing or non-reducing conditions (determined by the presence or absence of β-mercaptoethanol in the sample buffer, respectively). Unless specified, the studies presented in the text were performed under reducing conditions. For this study, we used 12% precast gels (Bio-Rad Laboratories, GmbH, Munich, Germany; #4561046). All the samples were analyzed at least in duplicate (duplicates in separate gels) and distributed in the gels to ensure the comparison by disease condition and *APOE* genotype. The distribution of the samples in the gels was performed by a member of the team and the experiments were performed by another, the experimenter, in a blind way.

Following electrophoresis, proteins were blotted onto 0.45-μm nitrocellulose membranes (Bio-Rad Laboratories, GmbH, Munich, Germany). Bands of apoE immunoreactivity were detected using either the antibody AB178479 (goat polyclonal; Merck Millipore) or the antibody AB947 (goat polyclonal; Merck Millipore), both common to all apoE isoforms, or alternatively by an antibody specific to the apoE4 isoform (recognizes an internal domain comprising the Arg112 residue present exclusively in apoE4 species; mouse monoclonal, Novus Biologicals; NBP1-49529). Blots were then probed with the appropriate conjugated secondary antibodies (IRDye secondary antibodies, LI-COR Biosciences, Lincoln, NE, USA) and imaged on an Odyssey CLx Infrared Imaging System (LI-COR Biosciences). Band intensities were analyzed using LI-COR software (ImageStudio Lite). The boxes selected with the ImageQuant Studio software for quantification, as well as the completed blots, are shown as supplementary figures. Recombinant apoE3 (Peprotech, ThermoFisher Scientific# 350-02) was included into each blot to serve as a loading reference and for normalizing the immunoreactivity signal between blots. Specifically, the same amount of recombinant apoE3 was always included, and the immunoreactivity of the apoE bands from each blot was referred to (divided by) the immunoreactivity of recombinant apoE3, thus correcting inter-blot differences and allowing for comparisons across assays.

For blue-native gel electrophoresis, the CSF samples were not heated (native conditions) and were loaded with NuPage LDS 4× Sample Buffer (ThermoFisher Scientific, NP007) into native-PAGE 4–16% gels (ThermoFisher Scientific, BN1002BOX). Buffers were prepared using native-PAGE Running Buffer (ThermoFisher Scientific, BN2001) and native-PAGE Cathode Buffer Additive (ThermoFisher Scientific, BN2002). Immunoreactivity was detected using the AB178479 antibody and HRP anti-goat secondary antibody (ThermoFisher). The signal was visualized by ECL (GE Healthcare Life Science) and analyzed using ImageStudio Lite.

### ApoE immunoprecipitation

CSF samples (50 μL) were incubated on a roller overnight with 100 μL PureProteome FlexiBind Magnetic Beads (Merck Millipore, LSKMAGN04) coupled with the AB178479 apoE antibody (Merck Millipore). The supernatant was removed, and the beads were washed and then resuspended and boiled at 98 °C for 5 min in SDS-PAGE sample buffer and analyzed by western blot with the AB947 antibody (Merck Millipore) or anti-apoE4 antibody (Novus Biologicals, NBP1-49529). For a control immunoprecipitation, beads were coupled with horse serum and then incubated with CSF samples.

### Enzymatic deglycosylation

Enzymatic deglycosylation was performed using an Agilent Enzymatic Deglycosylation Kit (Agilent Technologies, GK80110) following the manufacturer’s instructions. Briefly, for each condition, 30 μL of control or AD CSF was mixed with 10-μl incubation buffer and 2.5-μL denaturing buffer and heated at 100 °C for 5 min. The samples were then cooled down to room temperature, and 2.5 μL of detergent (15% NP-40) was added while mixing gently. O- (1 μL sialidase and 1 μL O-glycanase) or N-linked (1 μL N-glycanase) deglycosylating enzymes were then added according to each different condition (O-linked, N-linked, or O- and N-linked deglycosylation) and samples were heated at 37 °C for 3 h. Samples were then analyzed by western blot. As for control of the deglycosylation process, samples exposed to the same heating conditions but without deglycosylating enzymes were included.

### In-gel digestion

In-gel digestion was performed as previously described [33] in order to investigate the content of western blot immunoreactive bands of interest using an antibody-free method. Briefly, 1 mL of a pool of AD CSF (*APOE* ε3/ε4 and *APOE* ε3/ε4 cases) was immunoprecipitated with AB178479 antibody and loaded into SDS-polyacrylamide gel under reducing conditions, as described above. ApoE3 and apoE4 recombinant proteins (Peprotech, ThermoFisher Scientific# 350-02 and 350-04) were also loaded in the gel (10 pmol) and used as a reference for band excising and positive control. Upon electrophoresis, the gel was divided into two parts, one for protein visualization by SimplyBlue^TM^ SafeStain Coomassie (ThermoFisher Scientific, cat# LC6060) and one for blotting with the AB947 antibody as confirmation of band presence and location. Bands of interest were cut-out from the AD CSF gel lane and recombinant protein lanes and destained using a 1:1 mixture of acetonitrile and 50 mM ammonium bicarbonate solution twice for 15 min. Furthermore, gel pieces were de-hydrated with 100% acetonitrile and dried using a vacuum centrifuge. Samples were subsequently reduced with 10 mM dithiothreitol (DTT) for 1 h at 56 °C and alkylated with 25 mM iodoacetamide (IAA) for 45 min at room temperature in the dark. Gel pieces were further washed with 25mM ammonium bicarbonate, de-hydrated with 100% acetonitrile, and dried using a vacuum centrifuge once more. Samples were digested overnight at 37°C using 100 ng/μL trypsin enzyme (Sequencing Grade Modified Trypsin, #V511A, Promega). The next day, digestion was stopped by the addition of 2% trifluoroacetic acid and 75% acetonitrile solution, and peptides were collected into a new tube (Costar, #3207). Gel pieces were further extracted with the addition of 50% acetonitrile and 0.2% trifluoroacetic acid solution shaking for 30 min. The supernatant containing the peptides was transferred to the collection tube. Pooled extracts for each gel piece were dried through vacuum centrifugation and stored at −80 °C pending mass spectrometry (MS) analysis.

### Mass spectrometry data analysis

Dried in-gel digested samples were reconstituted in 7 μL 8% acetonitrile/8% formic acid solution and shaken for 30 min. A total of 6 μL of each sample was investigated using mass spectrometry (MS) analysis performed with a Dionex 3000 nanoflow liquid chromatography system coupled to a Q Exactive (both Thermo Fisher Scientific). Briefly, a reversed phase Acclaim PepMap C18 (100 Å pore size, 3 μm particle size, 20 mm length, 75 μm i.d., Thermo Fisher Scientific) trap column was used for online desalting and sample clean-up. Separation was performed with a reversed phase Acclaim PepMap RSLC C18 (100 Å pore size, 2 μm particle size, 75 μm i.d., 150 mm length, Thermo Fisher Scientific) column at a flow rate of 300 nL/min by applying a linear gradient of 0–40% B for 50 min. Mobile phase A was 0.1% formic acid in water (v/v) and mobile phase B was 0.1% formic acid and 84% acetonitrile in water (v/v/v).

Mass spectra were acquired in positive ion mode and in a data-dependent manner with a resolution setting of 70,000 for precursor and 17,500 for fragment ion acquisitions. Fragmentation was obtained by higher energy collision-induced dissociation (HCD) using a normalized collision energy (NCE) setting of 28. Database searches were made using PEAKS Studio XPRO (Bioinformatic Solutions, Inc., Waterloo, Canada).

### Statistical analysis

All the data was analyzed using GraphPad Prism (version 7; GraphPad software, San Diego, CA, USA). The test was used to analyze the distribution of each variable. Firstly, multiple comparisons were performed between groups, ANOVA was used for parametric variables, and the Kruskal-Wallis test for non-parametric variables. A Student’s *t*-test for parametric variables and a Mann-Whitney *U* test for non-parametric variables were employed for comparison between two groups and for determining precise *p* values. For correlations, the Pearson and Spearman tests were used. The results are shown as means ± SEM; the standard deviation (SD) and median values are also displayed as indicated in the figure legends.

## Results

### CSF apoE in Tg344-AD rats

**Fig. 1:**
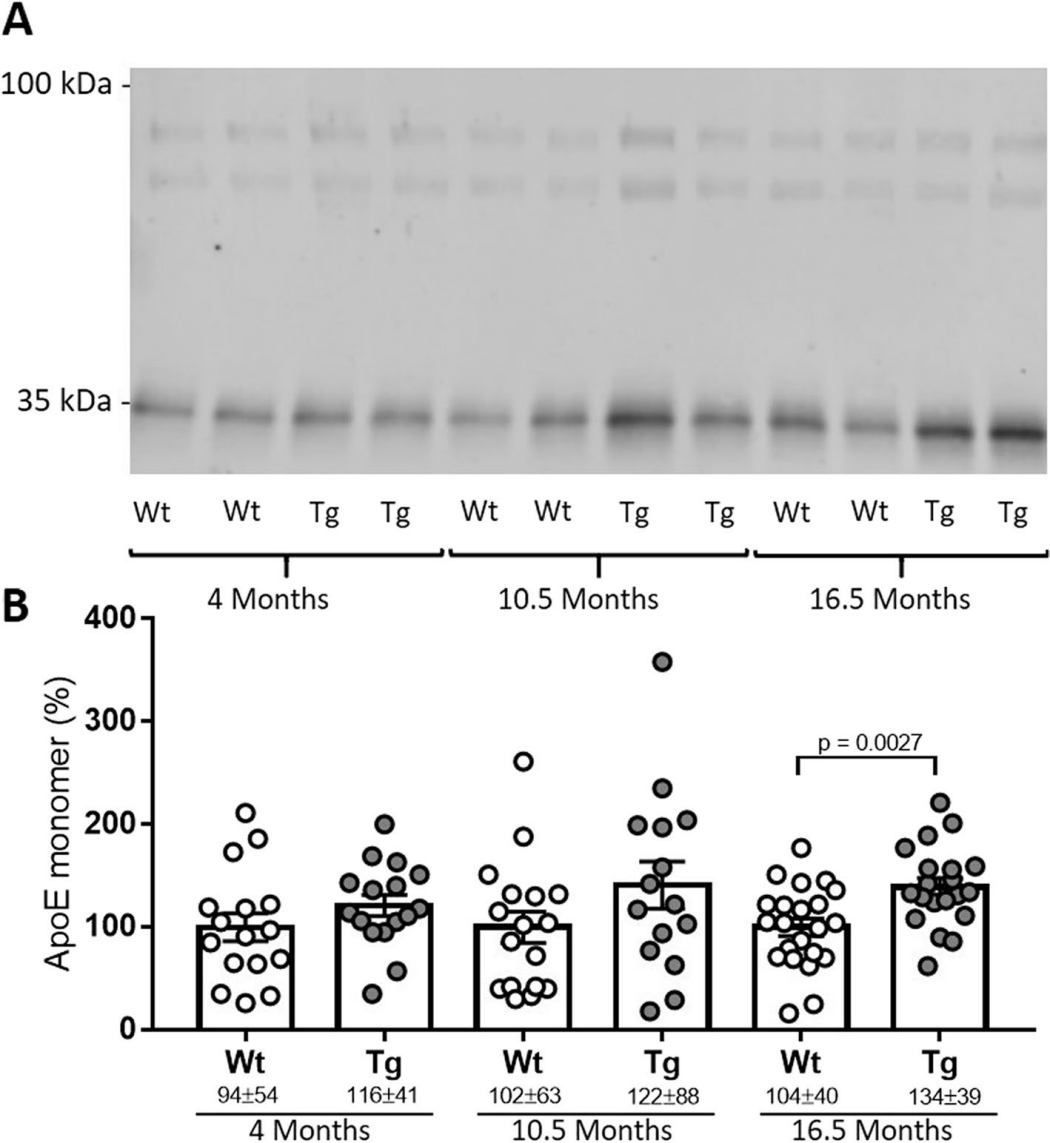
Analysis of CSF apoE in the TgF344-AD rats. **A** Representative blot of CSF obtained from wild-type (Wt) or transgenic (Tg) rats at 4, 10.5, and 16.5 months. The 100-kDa section of the blot presents enhanced contrast. **B** Quantification of apoE values obtained from western blots. Data is shown as a percentage with respect to the Wt values obtained at each age. The graphs represent mean ± SEM, and the numbers below represent median ± SD. A significant *p* value is indicated

To determine whether altered CSF apoE levels could be indicative of pathology-associated changes, we initially examined them in a rat transgenic AD model. The TgF344-AD rat expresses human *APP* with the Swedish mutation and human *PSEN1* with the Δ exon 9 mutation. As mentioned above, while humans have three versions of the *APOE* gene, other mammals such as rats only have one isoform of the apoE protein, which presents Arg at position 112 (https://web.expasy.org/variant_pages/VAR_000652.html) and thus shares the inability to form disulfide-linked dimers with human apoE4. We examined apoE levels in the CSF of 4-, 10.5-, and 16.5-month-old transgenic rats and wild-type littermates by SDS-PAGE using the AB178479 antibody. In all the animals and at all ages, apoE appeared as a single ~35-kDa band (Fig. 1A) and no differences were found between males and females (*p*> 0.05 for the comparison at every age). We did not find different glycoforms. Although no differences were found at 4 and 10.5 months between wild-type and TgF344-AD rats, a trend of apoE increment was observed. At 16.5 months of age, apoE levels were 50% higher in TgF344-AD animals than in wild-types (*p* = 0.003, Fig. 1B). The significant differences for CSF apoE levels detected between TgF344-AD and controls at 16.5 months of age were maintained when the animals were subgrouped by gender (male: control vs TgF344: *p* = 0.019; female: control vs TgF344: *p* = 0.048).

### Characterization of apoE in human CSF

We examined the presence of apoE species in CSF samples by SDS-PAGE and western blot under reducing conditions (in presence of the reducing agent β-mercaptoethanol that breaks disulfide bonds) from a cohort of control and AD patients from Gothenburg (Sweden; see Table 1) expressing different *APOE* genotypes.

**Fig. 2:**
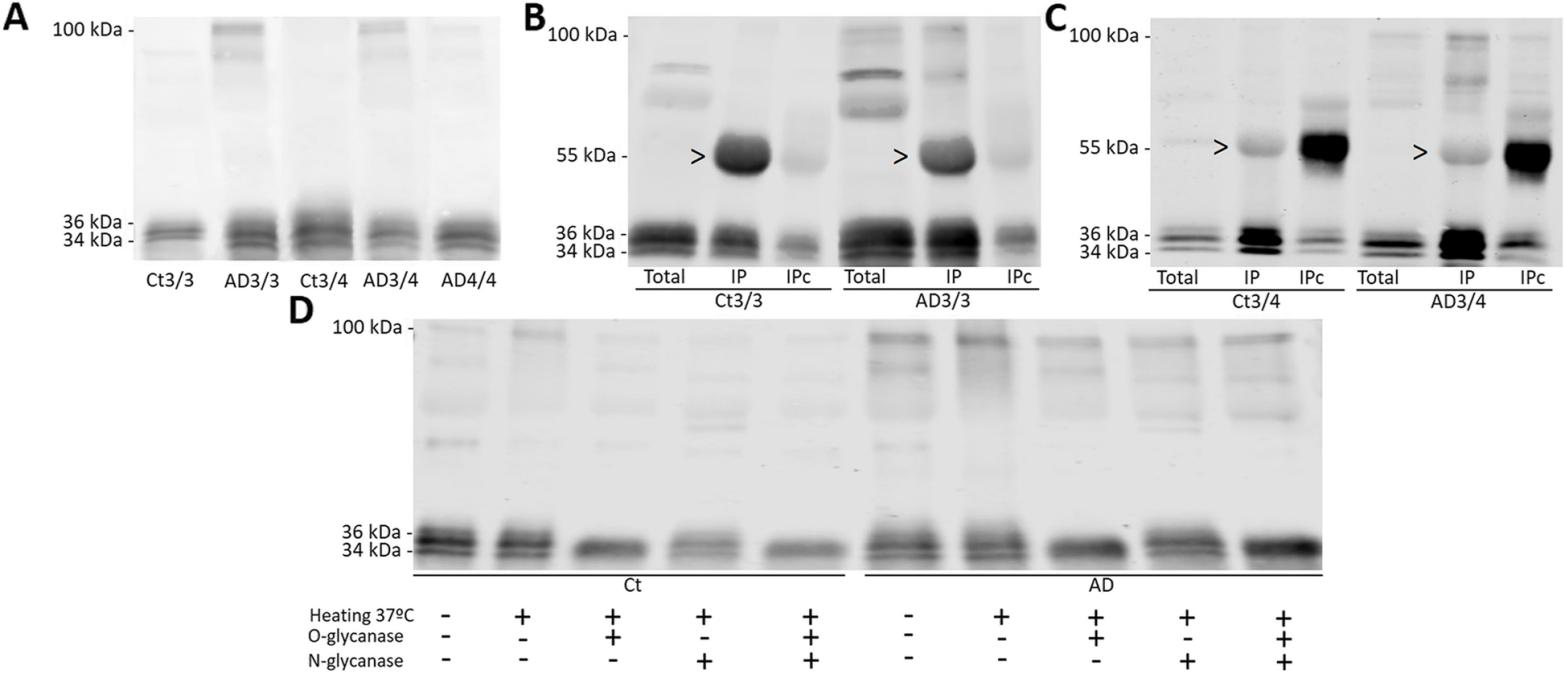
Characterization of apoE protein in human control (Ct) and AD CSF samples. **A** Representative immunoblot of CSF samples immunoblotted with apoE antibody (AB178479). CSF samples immunoprecipitated with the apoE AB178479 antibody (originated in goat) and immunoblotted with **B** apoE antibody AB947 (originated in goat) common to all apoE variants or **C** NBP1-49529 (originated in mouse), an antibody specific to the apoE4 isoform. Arrowheads: non-specific immunoglobulins (detected also in beads coupled with AB178479 in absence of human CSF; not shown). The bands detected between 34- and 36-kDa monomers and 100-kDa dimers were not consistently immunoprecipitated across trials. Total, CSF before IP; IP, immunoprecipitated protein; IPc, control immunoprecipitation. **D** CSF samples immunoblotted with apoE antibody (Ab178479) following O-linked, N-linked, or O- and N-linked deglycosylation. As a control of the deglycosylation process, samples under standard conditions and samples heated to 37 °C in the absence of deglycosylating enzymes were also included. Representative blots of three independent immunoprecipitation or deglycosylation experiments are shown

In all CSF samples, using the AB178479 antibody, apoE appeared as two distinct immunoreactive bands of ~34 and ~36 kDa (Fig. 2A). Immunoprecipitation with this antibody and subsequent immunoblotting with an alternative apoE antibody, AB947, confirmed the characterization of both apoE monomeric species in *APOE* ε3/ε3 samples (Fig. 2B). The same occurred when using an anti-apoE4 antibody in *APOE* ε3/ε4 samples (Fig. 2C). An apoE band of ~100 kDa was also observed, almost exclusively in the AD CSF samples, and this band was immunoprecipitated similarly to monomers (Fig. 2A–C). ApoE3 and apoE2 isoforms form disulfide-linked dimers in CSF, but these dimers should be sensitive to the reducing agent β-mercaptoethanol. Additional bands between the monomers and the 100-kDa bands did not appear to follow any specific pattern related with the pathology condition or the *APOE* genotype and were not consistently represented in the immunoprecipitated fraction; therefore, they were not considered for further investigations. Immunoprecipitated complexes of 100 kDa, using the AB178479 antibody, were dissected after electrophoresis and examined by MS analysis identifying 14 tryptic peptides spanning throughout the sequence of human apoE (Uniprot entry P02649_HUMAN), and both apoE3 and apoE4 isoforms were detected. Matching sequences are displayed in Table 2.

**Table 2:**
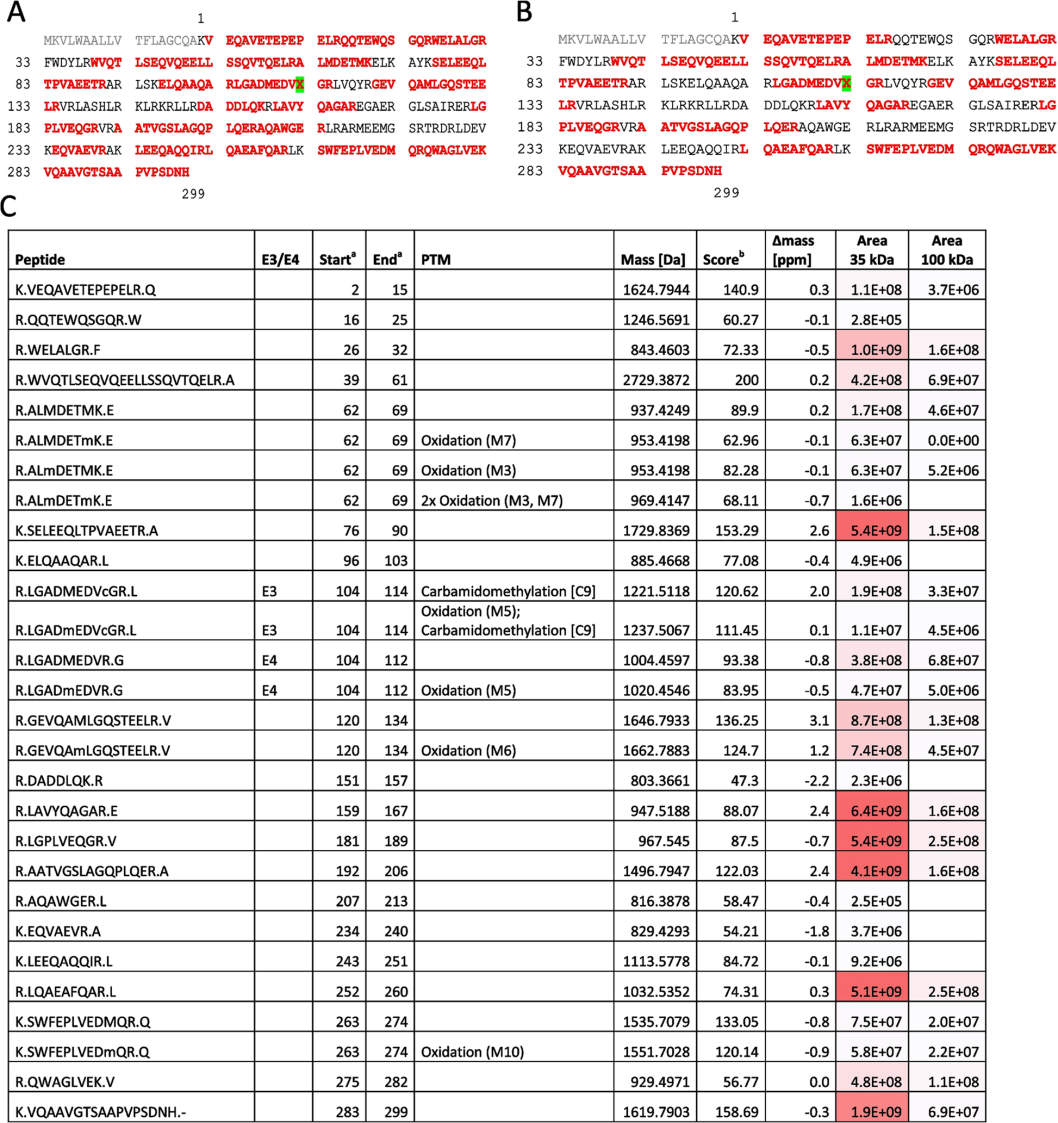
Identified peptides from apoE species of human CSF by MS

A, B: ApoE peptide chains assessed in monomeric (A) and the 100-kDa (B) species. The 18 aa signal peptide is shown in light gray; numbering is according to the mature protein, and X (marked in green) at position 112 denotes C (Cys) for ε3 and R (Arg) for ε4 isoforms, respectively. C: Data obtained from MS analysis. ^a^Start and end positions refer to the mature protein without signal peptide. ^b^Score as calculated by PEAKS Studio = −10 lg(*P*), where *P* is the probability for a false positive as determined by the software. *PTM* post-translational modifications, *ppm* mass error of the measured peptide in parts per million

We first aimed to understand why the monomers appeared as two distinct bands with different molecular masses. We hypothesized that the bands likely represent different glycoforms of the protein and, thus, an enzymatic deglycosylation assay was performed. While, as expected, N-deglycosylation did not alter the apoE band pattern, O-deglycosylation simplified the apoE pattern to a single immunoreactive band, suggesting that O-glycosylation could account for the differences in the molecular mass between the 36- and 34-kDa apoE species (Fig. 2D). The small differences in the electrophoretic mobility between glycosylated and deglycosylated apoE monomers are probably related to the slight carbohydrate mass associated to O-glycosylation, but also to changes in protein shape that affect their electrophoretic migration. Glycosylated proteins are normally more globular than non-glycosylated proteins, because the carbohydrate chains are not linear, even in reducing conditions. The apparent levels of the 100-kDa apoE band were not significantly modified following enzymatic deglycosylation, suggesting that these species are resistant to enzymatic deglycosylation (Fig. 2D). We observed the occurrence of apoE dimers in the recombinant protein (a non-glycosylated species since it is produced in bacteria), suggesting that sugar residues are not relevant epitopes for disulfide-linked dimer formation.

**Fig. 3:**
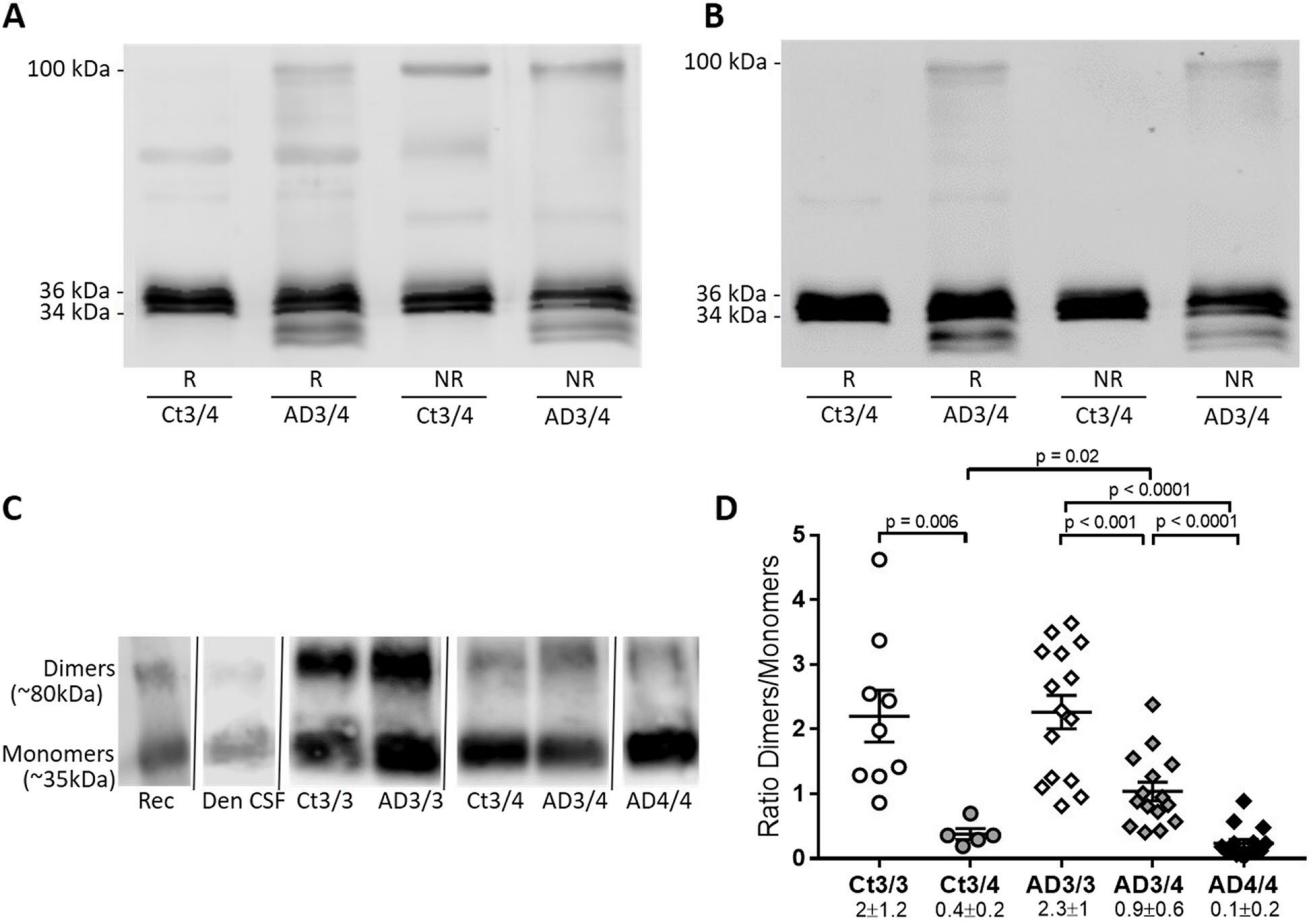
Characterization of CSF apoE species under reducing, non-reducing, and native conditions. **A**, **B** Control (Ct) and AD CSF samples immunoblotted under reducing (R) or non-reducing (NR) SDS-PAGE with **A** apoE antibody (Ab178479) or **B** apoE4-specific antibody (NBP1-49529). Note that apoE4 is identified as part of the 100-kDa bands despite the inability to form disulfide-linked dimers. **C** Representative blot of native-PAGE studies, showing the apoE monomers and dimers. The discrepancy in molecular weight is likely due to the differences between native-PAGE and SDS-PAGE electrophoretic conditions which affect the migration of proteins. Rec, recombinant apoE3; Den CSF, denatured CSF. **D** Quantification of the ratio of apoE dimers compared to monomers across the different *APOE* genotypes, estimated by native-PAGE (see **C**). Scatter plots of apoE levels are represented. The graphs represent mean ± SEM, and the numbers below represent median ± SD. Significant *p* values are indicated

As previously mentioned, the ~100-kDa apoE band was observed almost exclusively in AD CSF samples, including *APOE* ε4/ε4 samples (Fig. 2A). To further characterize this apoE species, we performed SDS-PAGE studies in non-reducing conditions to preserve the disulfide bonds (absence of β-mercaptoethanol). The 100-kDa apoE band in AD samples appeared to be indistinguishable from the one observed in reducing conditions, and remarkably, this band appeared in the control samples in non-reducing conditions, probably representing apoE dimers linked by disulfide bonds (Fig. 3A). When a specific antibody for apoE4 was used, NBP1-49529, the 100-kDa immunoreactivity was also detected in samples from *APOE* ε3/ε4 AD subjects under both reducing and non-reducing conditions, while no immunoreactivity was detected in *APOE* ε3/ε4 control samples under non-reducing conditions (Fig. 3B). This could corroborate that apoE4 in AD samples participates in complexes to form 100-kDa stable species, in both apoE3/4 or apoE4/4 subjects, that do not rely on disulfide bonds, due to its lack of Cys112, thus representing aberrant/anomalous apoE aggregates. In *APOE* ε3/ε4 control subjects, however, apoE4 does not participate in the 100-kDa species observed under non-reducing conditions, given its complete reliance on disulfide bonds to form functional dimers.

To determine the different contributions of the apoE 100-kDa species in AD and control cases, we first estimated the apoE dimer/monomer balance by native-PAGE electrophoresis. We included a CSF control sample under fully reducing and denaturing conditions, as well as an apoE3 recombinant protein under native conditions, which served to identify the monomeric and dimeric apoE bands. Two immunoreactive apoE bands were observed, likely representing apoE monomers and dimers (Fig. 3C). An immunoreactive band compatible with dimeric complexes was detected in CSF from AD *APOE* ε4/ε4 cases, whose lack of Cys112 should eliminate their ability to form disulfide-bond-dependent complexes. Given the difficulty of finding age-matched *APOE* ε4/ε4 control subjects (low prevalence of this genotype in the general and healthy population), we cannot compare AD *APOE* ε4/ε4 with control ε4/ε4 cases. We compared the apoE dimer/monomer quotient (ratio D/M) between AD CSF samples and controls, subgrouping the samples by *APOE* genotype (Fig. 3D). In the control group, the dimer/monomer quotient was significantly lower in the *APOE* ε3/ε4 group (ratio D/M = 0.38) compared to that of the ε3/ε3 group (ratio D/M = 2.20; *p*= 0.006), associated to the inability of the apoE4 isoform to form disulfide-linked dimers. The same situation was found in the AD group, where the dimer/monomer ratio decreased as the ε4 allele was present (ε3/ε3: ratio D/M = 2.27; ε3/ε4: ratio D/M = 1.04; ε4/ε4: ratio D/M = 0.24). For *APOE* ε3/ε3 subjects, the dimer/monomer ratio in controls was not significantly different to that found in AD subjects; however, for *APOE* ε3/ε4 subjects, the quotient was higher in the AD group compared with controls (*p* = 0.02; Fig. 3D). This may be reflecting the accumulation of aberrant aggregates in the AD samples expressing apoE4, in addition to the physiological disulfide-bound dimers present in controls.

### Levels of CSF apoE species in AD

**Fig. 4:**
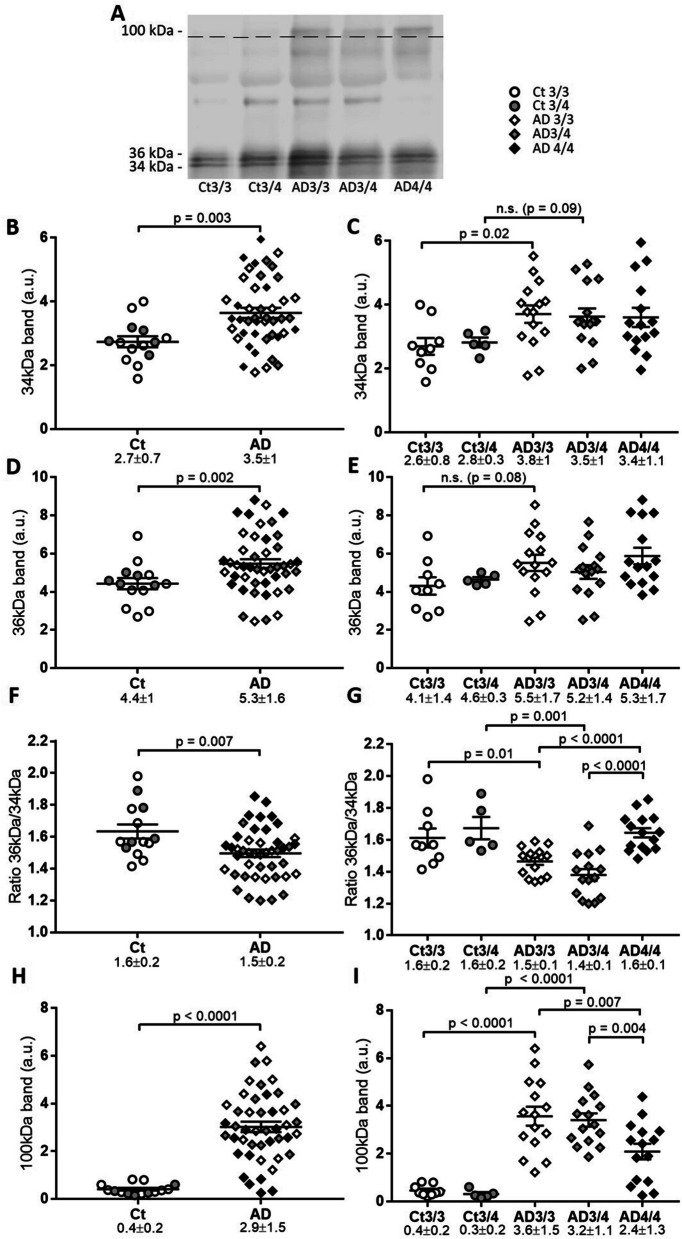
Analysis of CSF apoE species from the Gothenburg cohort. Control (Ct) and AD CSF samples analyzed by SDS-PAGE. Each individual band was quantified and normalized to the reference value (recombinant apoE). **A** Representative immunoblot of CSF samples with apoE antibody and legend for graphs. The 100-kDa section of the blot presents enhanced contrast. **B**, **C** Statistical analysis of the 34-kDa apoE immunoreactive band in **B** control and AD and by **C***APOE* genotype. **D**, **E** Statistical analysis of the 36-kDa apoE immunoreactive band in **D** control and AD and by **E***APOE* genotype. **F**, **G** Statistical analysis of the ratio of 36-kDa/34-kDa immunoreactive bands in **F** control and AD and by **G***APOE* genotype. **H**, **I** Statistical analysis of the 100-kDa apoE immunoreactive band in **H** control and AD and by **I***APOE* genotype. The graphs represent mean ± SEM, and the numbers below represent median ± SD. Significant *p* values are indicated

Given the differences in CSF apoE aggregates found under native conditions between AD and controls, we evaluated the levels of the 34-kDa, 36-kDa, and 100-kDa apoE species by SDS-PAGE in reducing conditions and western blotting, using the AB178479 antibody (Fig. 4A). The 34-kDa apoE band was significantly increased in AD compared with controls (*p* = 0.003; Fig. 4B). When discriminating by *APOE* genotype, only the *APOE* ε3/ε3 genotype was significantly elevated in AD compared with control samples (*p* = 0.02; Fig. 4C). The same analysis within the *APOE* ε3/ε4 genotype exhibited less statistical power because the size of the control group is small; nonetheless, we observed a trend of 34-kDa apoE increment in ε3/ε4 genotype AD samples with respect to controls (*p* = 0.09; Fig. 4C). The 36-kDa apoE appeared significantly increased in AD compared with controls overall (*p* = 0.002; Fig. 4D), but not among *APOE* genotypes (Fig. 4E).

As expected, when we considered the sum of the apoE immunoreactivity for the 34- and 36-kDa bands, increased levels were seen in AD patients (27 ± 5%), as compared with controls (*p* = 0.005). Despite the fact that these results indicate a net increase of CSF apoE in AD samples, when defining a quotient between the apoE monomeric glycoforms (ratio 36 kDa/34 kDa) we detected an imbalance in the AD samples, which displayed a decreased 36-kDa/34-kDa ratio compared with controls (*p* = 0.007; Fig. 4F). These differences were maintained when the samples were separated by *APOE* genotype (ε3/ε3 control: ratio 36 kDa/34 kDa = 1.61 vs ε3/ε3 AD: ratio 36 kDa/34 kDa = 1.46, *p* = 0.01; ε3/ε4 control: ratio 36 kDa/34 kDa = 1.67 vs ε3/ε4 AD: ratio = 1.38, *p* = 0.001; Fig. 4G). Within the AD group, we also observed significant differences between the genotypes, as the 36-kDa/34-kDa ratio was significantly lower in *APOE* ε3/ε3 (*p* < 0.0001) and ε3/ε4 (*p* < 0.0001) samples when compared with ε4/ε4 AD samples (ratio 36 kDa/34 kDa = 1.64). In each group, CSF apoE levels appeared unaltered when subgrouping between males and females (*p* > 0.05 for all the subgroups). There were no clear correlations between the level of the 34- or 36-kDa apoE or the 36-kDa/34-kDa ratio with the age of the subjects, in either of the groups considered individually.

The immunoreactivity of the 100-kDa apoE species was quite faint in control samples, and accordingly, substantial differences were found between AD samples and controls (*p* < 0.0001; Fig. 4H, I). In the AD group, the 100-kDa apoE species levels were significantly lower in the *APOE* ε4/ε4 group when compared to both ε3/ε3 (*p* < 0.0001) and ε3/ε4 (*p* < 0.0001) groups. Interestingly, in the *APOE* ε4/ε4 AD cases, the samples with the lowest 100-kDa apoE immunoreactivity belonged to the youngest subjects (*n* = 5, 68±2 years), as compared to the other cases (*n*= 10, 76±1 years; *p*=0.002). Within the AD *APOE* ε4/ε4 subgroup, we also observed a statistically significant positive correlation between the age of participants and the immunoreactivity of the 100-kDa apoE band (*r* = 0.622, *p* = 0.013). For the rest of the groups, we failed to determine a correlation between the 100-kDa band and age.

Interestingly, the quotient of 36 kDa/34 kDa monomeric glycoforms (*r* = 0.40, *p* = 0.007), as well as the levels of the 34-kDa species (*r* = 0.32, *p* = 0.034), correlated with Aβ42 in AD individuals. Meanwhile, the levels of the 100-kDa apoE complexes correlated with T-tau levels (*r* = 0.33, *p* = 0.028), yet failed to achieve significance with the Aβ42 levels (*r* = 0.27, *p* = 0.070).

### Levels of CSF apoE species in AD in a second cohort

**Fig. 5:**
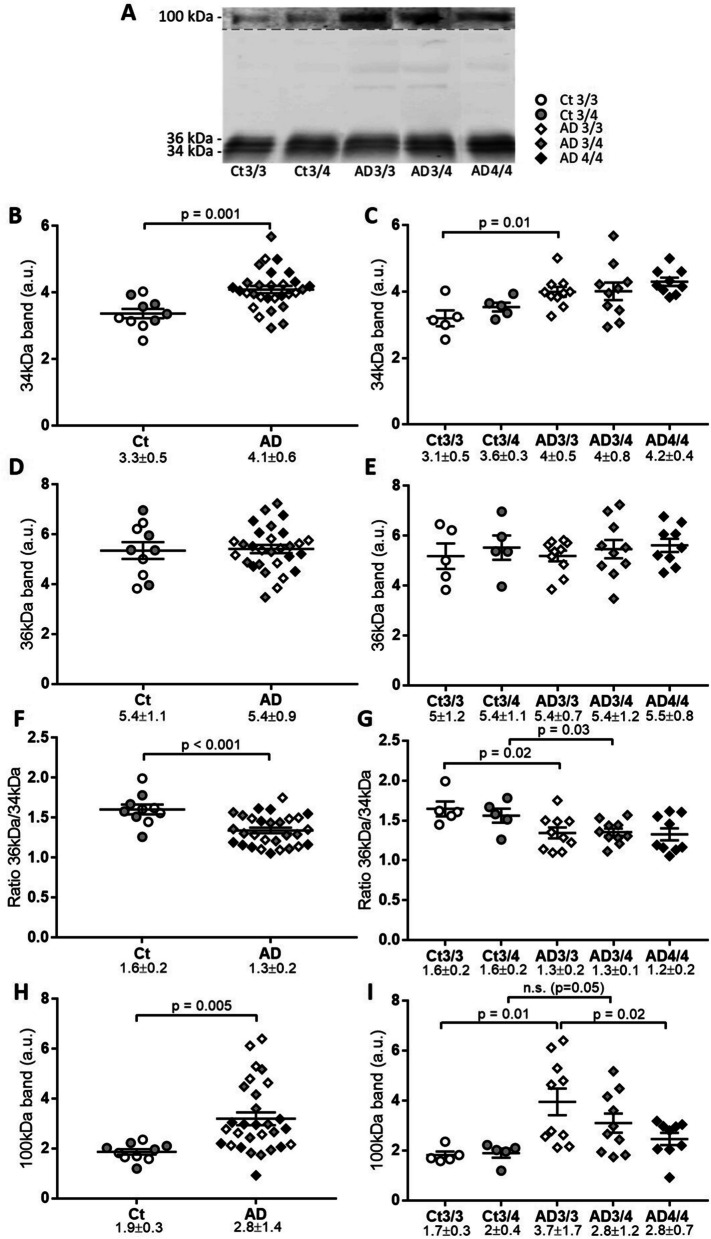
Analysis of CSF apoE species from the Barcelona cohort. Control (Ct) and AD CSF samples analyzed by SDS-PAGE. Each individual band was quantified and normalized to the reference value (recombinant apoE). **A** Representative immunoblot of CSF samples with apoE antibody and legend for graphs. The 100-kDa section of the blot presents enhanced contrast. **B**, **C** Statistical analysis of the 34-kDa apoE immunoreactive band in **B** control and AD and by **C***APOE* genotype. **D**, **E** Statistical analysis of the 36-kDa apoE immunoreactive band in **D** control and AD and by **E***APOE* genotype. **F**, **G** Statistical analysis of the ratio of 36-kDa/34-kDa immunoreactive bands in **F** control and AD and by **G***APOE* genotype. **H**, **I** Statistical analysis of the 100-kDa apoE immunoreactive band in **H** control and AD and by **I***APOE* genotype. The graphs represent mean ± SEM, and the numbers below represent median ± SD. Significant *p* values are indicated

We attempted to validate our results in a second independent cohort of CSF samples from Barcelona (see Table 1, Fig. 5A). As in the first cohort, the analyses were performed by SDS-PAGE under reducing conditions. In this cohort, the 34-kDa apoE levels were significantly higher in AD compared with controls (*p=*0.001), and specifically only for those with *APOE* ε3/ε3 genotype (*p* = 0.01) (Fig. 5B, C). In contrast to the first cohort, no differences in the 36-kDa species were detected (Fig. 5D, E). The 36-kDa/34-kDa ratio was again lower in AD compared with control samples (*p* < 0.001, Fig. 5F), and this difference was maintained when the samples were separated by genotype (AD vs controls for *APOE* ε3/ε3, *p*=0.02, and for *APOE* ε3/ε4, *p*=0.03) (Fig. 5G). ApoE levels once again appeared to be unaltered when subgrouping between males and females (*p*> 0.05 for all comparisons). In this cohort, we also failed to correlate 34- or 36-kDa levels or the 36-kDa/34-kDa ratio with the age of the subjects.

As in the first cohort, the 100-kDa apoE levels were higher in AD than in controls (*p* = 0.005; Fig. 5H). When the samples were stratified by *APOE* genotype (Fig. 5I), the significant differences between AD and controls were maintained in the ε3/ε3 group (*p* = 0.01) and were in the limit of statistical significance in the *APOE* ε3/ε4 group (*p* = 0.050) despite the small number of controls. Within the AD group, the *APOE* ε4/ε4 samples once again displayed significantly lower 100-kDa apoE levels compared with ε3/ε3 cases (*p* = 0.02). The results from this cohort corroborate that 100-kDa apoE is associated to AD and that apoE4 has a reduced capacity to form these complexes.

In this cohort, and exclusively in the AD group overall, we detected a significant correlation between the age of the subjects and the 100-kDa apoE species (*r* =0.645, *p*= 0.0002), indicating that the appearance of the aberrant apoE aggregates may be related to aging in AD. This association was maintained within the *APOE* ε3/ε3 (*r* = 0.813, *p*= 0.004) and the ε3/ε4 (*r* = 0.799, *p*= 0.006) AD groups.

In this cohort, only the levels of the 100-kDa apoE species correlated with Aβ42 levels (*r*= 0.41, *p*= 0.027). Thus, despite finding some correlations, none of these significant correlations resulted in consistency between the two cohorts.

## Discussion

Typically, transgenic models produce pathological changes that partially replicate changes seen in human patients. In this study, firstly, we have found an increase in CSF apoE in the TgF344-AD rats, with the documented occurrence of amyloid pathology around 10 months of age [31, 34]. This result can be interpreted as a suggestive gain of function for apoE in AD. In fact, this increase in CSF apoE content is similar to the one observed in AD patients when considering total apoE content. Considering the summation of the apoE immunoreactivity for 34- and 36-kDa (not including the value for the 100-kDa band) species, in samples from AD patients, a significant overall increase in total CSF apoE was found in the Gothenburg cohort and a non-significant trend to increase was seen in the Barcelona cohort compared to controls. However, the biochemical discrimination of different human CSF apoE species and the altered balance of these species lead us to believe that, despite the increase in total CSF apoE levels determined in the AD transgenic model and AD patients, the imbalance between apoE species should be interpreted as indicative of a potential impairment in apoE function in the brain. Thus, higher levels of apoE could paradoxically result in less functionality if the increase is represented by complexes and immature glycoforms.

Indeed, we have identified two monomeric apoE species in human CSF and demonstrated that the balance between these species in AD patients differs compared to that of controls. Some previous studies that did not distinguish the contribution of particular apoE species have indicated that CSF apoE levels in AD patients are increased [35], also at follow-up [36], but many studies addressing total CSF apoE levels are inconclusive and found no clear association with the AD condition or *APOE* genotype [20–22]. In addition to recurrent confounding factors such as the handling of the samples, and also considering differences in the diagnostic accuracy between cohorts, the inconsistencies found in these previous reports could be associated mostly with the determination method used, as some are based in MS [17, 18], while others use immunoassays [16], both of which fail to discriminate between apoE species. Even if an immunoassay is the most available and desirable approach for quantitative analysis of altered levels of a biomarker, this method does not easily detect subtle changes in specific species (imbalance in glycoforms) and/or does not detect particular species suffering conformational changes (aberrant dimers).

The 34- and 36-kDa species are likely different O-glycoforms, and the difference in electrophoretic mobility of the apoE glycoforms could be a consequence of its sialylation [37]. ApoE is exclusively O-glycosylated and can be capped with one or two sialic acids [5]. In CSF, the existence of two glycans per molecule of apoE has been demonstrated [38], and previous studies indicate that astrocytes secrete two differential glycoforms of apoE [39] and that the sialo and asialo forms of apoE can both be secreted into the medium [40]. Our results indicate that the 34-kDa apoE monomers, which appear to be less sialylated than 36-kDa apoE monomers [3], are present at a higher proportion in AD subjects compared with controls, in both independent cohorts. Whether or not these 34-kDa species can participate in disulfide-linked apoE dimers or pathological complexes, as described here, requires further study.

Moreover, the altered balance between apoE glycoforms should be validated in external cohorts. Here, most of the results obtained in the Gothenburg cohort were validated in a second independent cohort from Barcelona, despite the small size of the groups in this cohort. Nonetheless, some inconsistent results were observed between cohorts regarding the ratio of the 36-kDa/34-kDa species. In the Gothenburg cohort, this ratio was significantly higher in AD individuals with an *APOE* ε4/ε4 genotype compared with *APOE* ε3/ε3 and ε3/ε4, while in the Barcelona cohort, the ratios were at a similar level among AD individuals with different *APOE* genotypes. Additional studies will serve to determine if the imbalance between apoE glycoforms is a common feature for AD *APOE*-ε4 homozygote subjects.

Indeed, the changes observed in this study are less obvious in ε4/ε4 samples. This discrepancy may be due to the fact that small changes in apoE levels for ε4/ε4 subjects could be more detrimental than in the rest of *APOE* genotypes, perhaps caused by the basal compromise in some of the biological functions of apoE in the brain related with the inability of the apoE4 isoform to form dimers.

*APOE-*ε4 is the strongest risk factor gene for AD, although inheriting *APOE-*ε4 does not mean a person will definitely develop the disease. Thus, the opportunity to analyze the subset of *APOE* ε3/ε4 control individuals with no AD-like symptoms is very interesting. As stated, all the cases were retrospectively selected from large cohorts and based on the determination of AD core biomarkers. The diagnostic uncertainty is inherent in this type of studies, but the control individuals with *APOE* ε3/ε4 genotype displayed similar apoE values as the ones obtained in *APOE* ε3/ε3 individuals.

Correct apoE glycosylation is fundamental for its function and lipoprotein binding capacity. ApoE glycosylation can modulate receptor affinity, lipid-binding ability, lipid transportation, and metabolic functions [41–43]. Furthermore, apoE deglycosylation reduces its binding to Aβ42 [44] and may induce Aβ42 accumulation [45]. Our results suggest that the imbalance between the different glycoforms of apoE monomers observed in AD may interfere with its biological function, contributing to the progression of the disease. Interestingly, apoE glycosylation also plays a key role in the protection against self-association and spontaneous aggregation [46].

As mentioned, the apoE isoforms encoded by *APOE* ε3 or ε2 are able to form disulfide-linked hetero- and homodimers through the Cys residue at position 112, while *APOE* ε4 (which presents Arg at position 112) and apoE from non-human mammals are unable to form these oligomeric species. However, in our studies, apoE4 isoforms were present in 100-kDa aggregates in *APOE* ε3/ε4 AD cases, and these aggregates were identified in most of the *APOE* ε4/ε4 AD patients. These 100-kDa complexes are compatible in molecular mass to disulfide-linked apoE dimers, which exist as a major portion of apoE in human CSF of *APOE* ε3 or ε2 carriers [16]. The existence of SDS-resistant dimers of apoE4 was suggested when studying the in vitro formation of SDS-resistant Aβ-apoE complexes [47]; but, to our knowledge, it has never been demonstrated in vitro or in vivo. The definitive identity of the 100-kDa species was confirmed by the diverse immunoprecipitation analyses combining antibodies originated from diverse animal species and the MS studies. Rats express a unique apoE variant most closely related to the human ε4-type haplotype. However, in the transgenic rat model of AD, we were not able to observe the 100-kDa resistant apoE species that we observed in AD *APOE* ε4/4 cases. Likewise, the possibility that inactive monomers of apoE occur in this animal model requires further study; however, models in which the amyloid condition results in an increase of apoE expression should consider this possibility.

ApoE dimers or multimers may be the biologically important species, particularly in receptor binding [15]. In a previous study, the levels of apoE dimers in the CSF from AD subjects were not different from those in controls [48], although in this study they did not assess the nature of the aberrant β-mercaptoethanol resistant complexes. In our AD samples, the 100-kDa apoE complexes are aberrantly resistant to reducing conditions; thus, they may represent a different species compared to the biologically active disulfide-bound dimers. The relevance of an apoE dimer/monomer profile in AD was also addressed previously in plasma, with the identification of dimers only in *APOE*-ɛ3 carrier subjects, the levels of which decreased in the demented group [49]. A recent report using two-dimensional gel electrophoresis indicated that plasma apoE is elevated in AD with respect to controls [50]. However, it is worth noting that apoE does not cross the blood-CSF barrier [51].

ApoE can form heteromeric complexes with other apolipoproteins [17] and with proteins such as the ciliary neurotrophic factor [52] or APP [53], among others, but principally with Aβ. Indeed, apoE can form in vitro SDS-stable complexes with Aβ [1, 54, 55], but the interaction with exogenous Aβ does not induce drastic changes to the overall size of the Aβ/apoE-containing lipoprotein particles [55]. The formation of noncovalent apoE/Aβ complexes (1:1) is implicated in both Aβ clearance and fibrillization, and the three isoforms of apoE are able to form these complexes [56]. Complexes of apoE and Aβ have been demonstrated in non-pathological human CSF [55] and in AD brain [57, 58]. Thus, Aβ may act as a triggering driver for the crosslinking and stabilization of aberrant apoE complexes. In the AD brain, the balance between soluble to insoluble apoE/Aβ aggregates has been associated with impaired apoE activity in Aβ clearance, as apoE is responsible for the accumulation and fibrillization of Aβ [59]. The effects of apoE on Aβ aggregation may be restricted to HDL-like particle-bound apoE [60]. Other studies have demonstrated that apoE influences Aβ clearance despite minimal interaction [61]. However, despite the fact that Aβ can contribute to the formation of stable apoE dimers as a crosslinking agent, the behavior of the resulting species may differ from other apoE/Aβ aggregates. We favor the hypothesis that the stable apoE complexes may have compromised biological activity, regardless of the presence of Aβ.

It is also interesting to note that apoE binds Aβ in an isoform-specific manner. Thus, monomeric apoE4 binds to Aβ peptide more rapidly than monomeric apoE3 or apoE2, and so it appears that the efficiency of binding correlates inversely with the risk of developing AD pathology [62]. Moreover, soluble SDS-stable complexes of apoE4/Aβ precipitate more rapidly than apoE3/Aβ complexes [63]. Whether these monomeric apoE/Aβ complexes trigger the formation of oligomeric complexes, and the potential compromise of the apoE peptides involved in these complexes on Aβ clearance in vivo, require analysis.

The aberrant apoE complexes may also influence the role of apoE on lipid metabolism and transport. It is assumed that unlipidated apoE monomers are the species that form disulfide-linked dimers; however, it is also believed that apoE must be properly lipidated to participate in cholesterol and lipid transport. Aberrant dimers are not linked by disulfide bonds, but we can only speculate whether these species are lipidated or not, and if the occurrence of these aberrant dimers could compromise the role of apoE regulating lipid homeostasis by mediating lipid metabolism and transport. ApoE4 is poorly lipidated compared with apoE2 and apoE3 [64], and reduced binding affinity of apoE4 for HDL results in a greater proportion of unlipidated apoE, hence forming aggregates that can be more toxic for neurons than apoE2 and apoE3 aggregates [65]. Since lipidation of apoE impedes aggregate formation [66], we presume that these aberrant dimers are not lipidated; nonetheless, this possibility should be tested.

Finally, we found a correlation between the 100-kDa apoE levels and age in AD samples, which suggests that during pathological aging, apoE could be more likely to form non-disulfide-bound aggregates in the CSF. In the TgF344 rats, only the older animals showed statistically significant high apoE levels; accordingly, these AD models show an age-dependent increase of the levels of Aβ40 and Aβ42 from 6 months of age [31].

The imbalance of apoE glycoforms and the existence of aberrant apoE aggregates in the CSF from AD individuals could be considered as a read-out of alterations of the biological activity of apoE in the brain of AD individuals. The possibility that CSF levels of apoE are under strong genetic influence by the *APOE* polymorphism is plausible; however, the relevance of these changes in CSF apoE levels on AD pathology remains elusive. The net increase of apoE levels in the CSF from AD individuals could be favored by aging. This increment, mainly due to the 34-kDa glycoform of apoE, which is likely hypo-sialylated, and the appearance of a β-mercaptoethanol-resistant 100-kDa apoE species, could indicate that the ability of apoE in AD to achieve its biological functions may be compromised.

In conclusion, while apoE levels tend to increase in AD CSF, this increase is more noticeable in certain glycoforms of monomers and aberrant complexes that may hinder its biological activity. A specific description of how these species affect apoE signaling and Aβ clearance should improve our understanding of the role of apoE in the AD pathology.

## Conclusions

The imbalance of apoE glycoforms and the existence of aberrant apoE aggregates in the CSF from AD individuals could be considered as a read-out of alterations of the biological activity of apoE in the brain of AD individuals. The possibility that CSF levels of apoE are under strong genetic influence by the *APOE* polymorphism is plausible; however, the relevance of these changes in CSF apoE levels on AD pathology remains elusive. The net increase of apoE levels in the CSF from AD individuals could be favored by aging. This increment, mainly due to the 34-kDa glycoform of apoE, which is likely hypo-sialylated, and the appearance of a β-mercaptoethanol-resistant 100-kDa apoE species, could indicate that the ability of apoE in AD to achieve its biological functions may be compromised.

In conclusion, while apoE levels tend to increase in AD CSF, this increase is more noticeable in certain glycoforms of monomers and aberrant complexes that may hinder its biological activity. A specific description of how these species affect apoE signaling and Aβ clearance should improve our understanding of the role of apoE in the AD pathology.

## Supplementary Information

**Additional file 1.** Images of complete blots from which figures were obtained, and also the boxes selected with the ImageQuant Studio software for quantification.

## Data Availability

The datasets used and/or analyzed during the current study are available from the corresponding author on reasonable request.

## Abbreviations

Aβ: Amyloid-beta
AD: Alzheimer’s disease
APP: Amyloid precursor protein
ApoE: Apolipoprotein E
CSF: Cerebrospinal fluid
CNS: Central nervous system
HDL: High-density lipoprotein
MS: Mass spectrometry
SDS: Sodium dodecyl sulfate
PAGE: Polyacrylamide gel electrophoresis
Tg: Transgenic
Wt: Wild-type
