# Supplementary material for: Apolipoprotein E imbalance in the cerebrospinal fluid of Alzheimer’s disease patients
Source: Alzheimers Res Ther. 2022 Nov 2;14:161. doi: 10.1186/s13195-022-01108-2 (PMC9628034; doi:10.1186/s13195-022-01108-2)
Supplement: Supplementary file 1 — Additional file 1. Images of complete blots from which figures were obtained, and also the boxes selected with the ImageQuant Studio software for quantification. [file 13195_2022_1108_MOESM1_ESM.pdf]

Figure 1A. ApoE in rat CSF

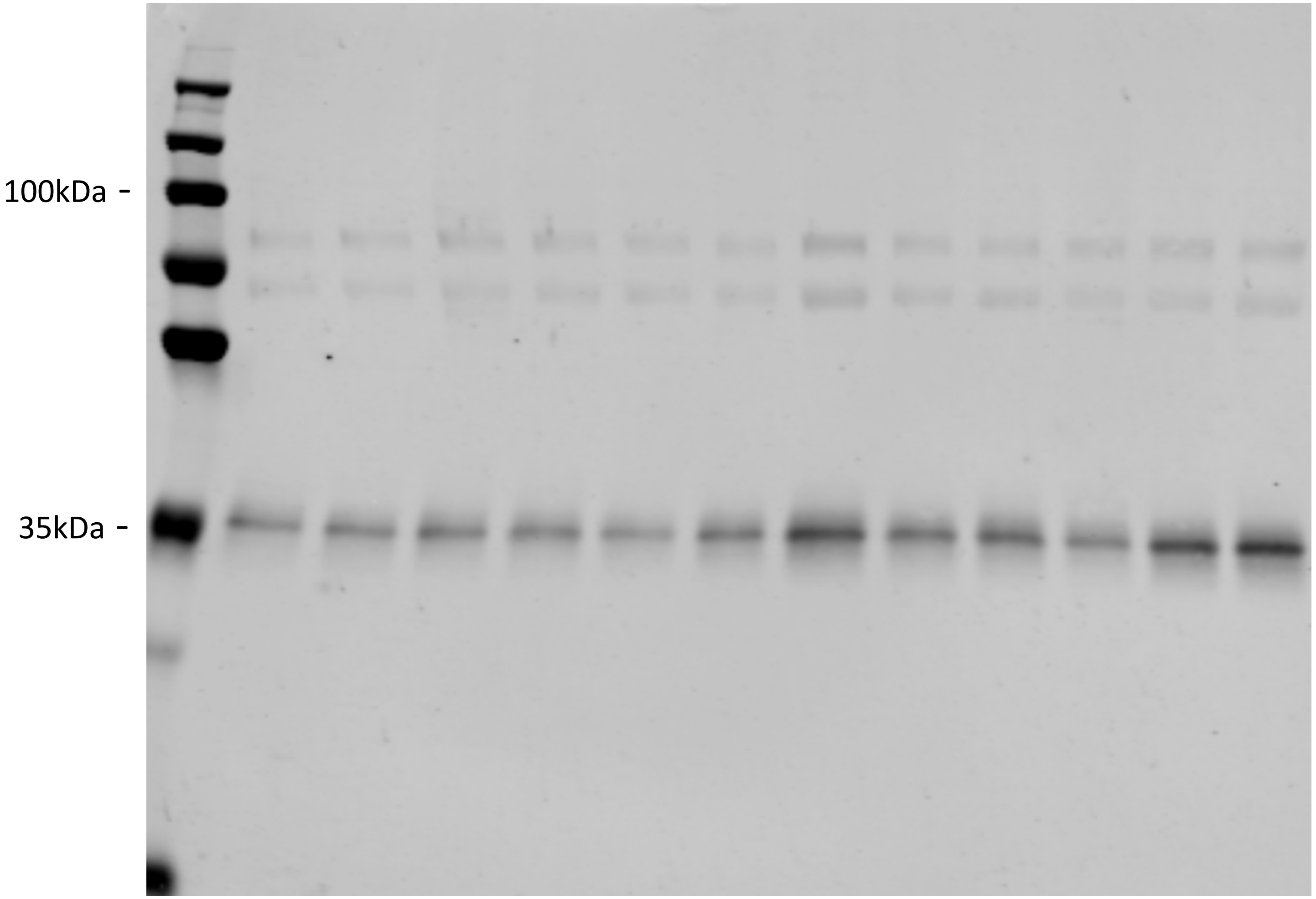

Figure 2A. ApoE in human CSF

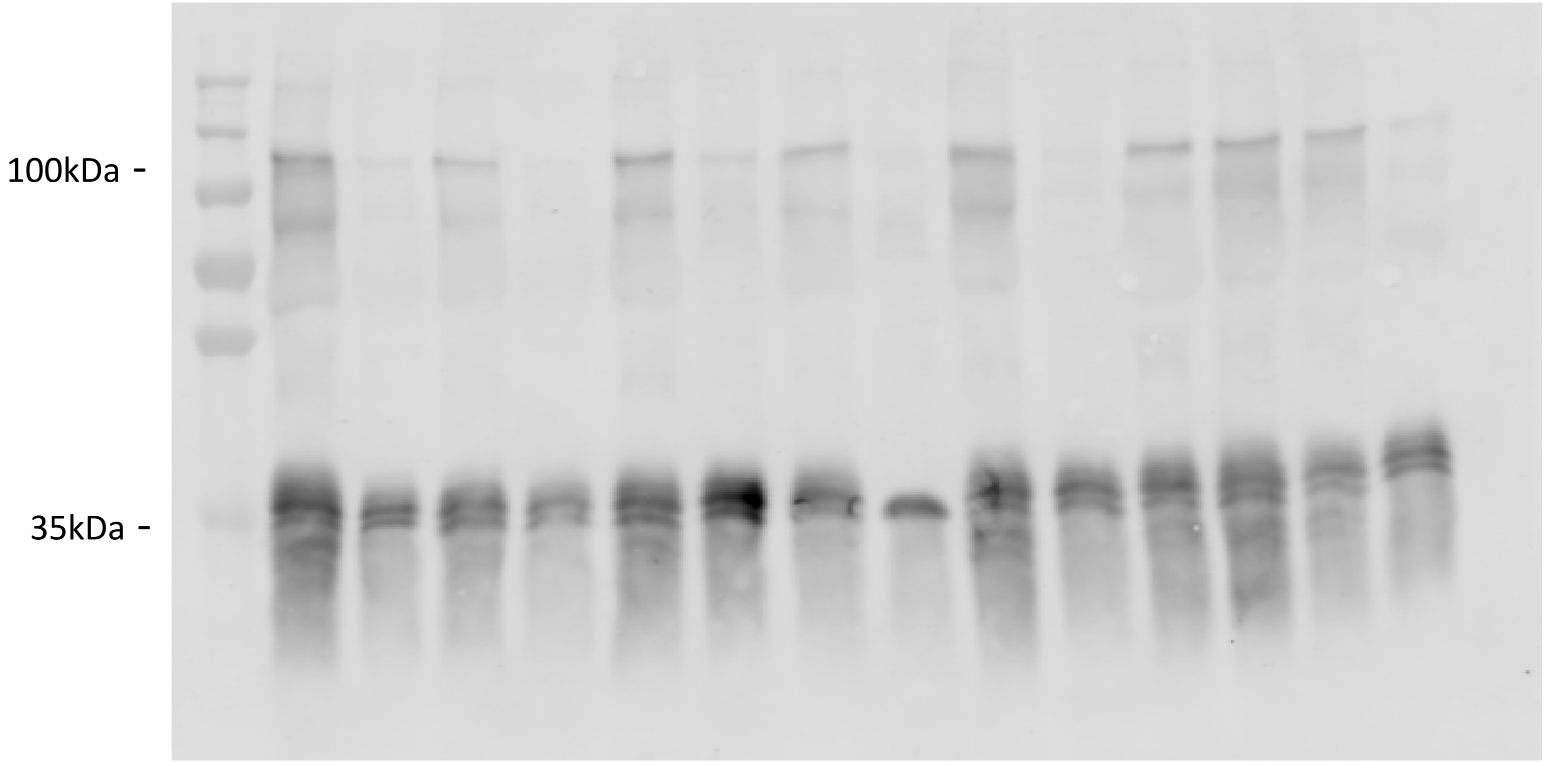

Lanes 3, 4, 11, 14 and 15

Figure 2B. ApoE immunoprecipitation in human CSF

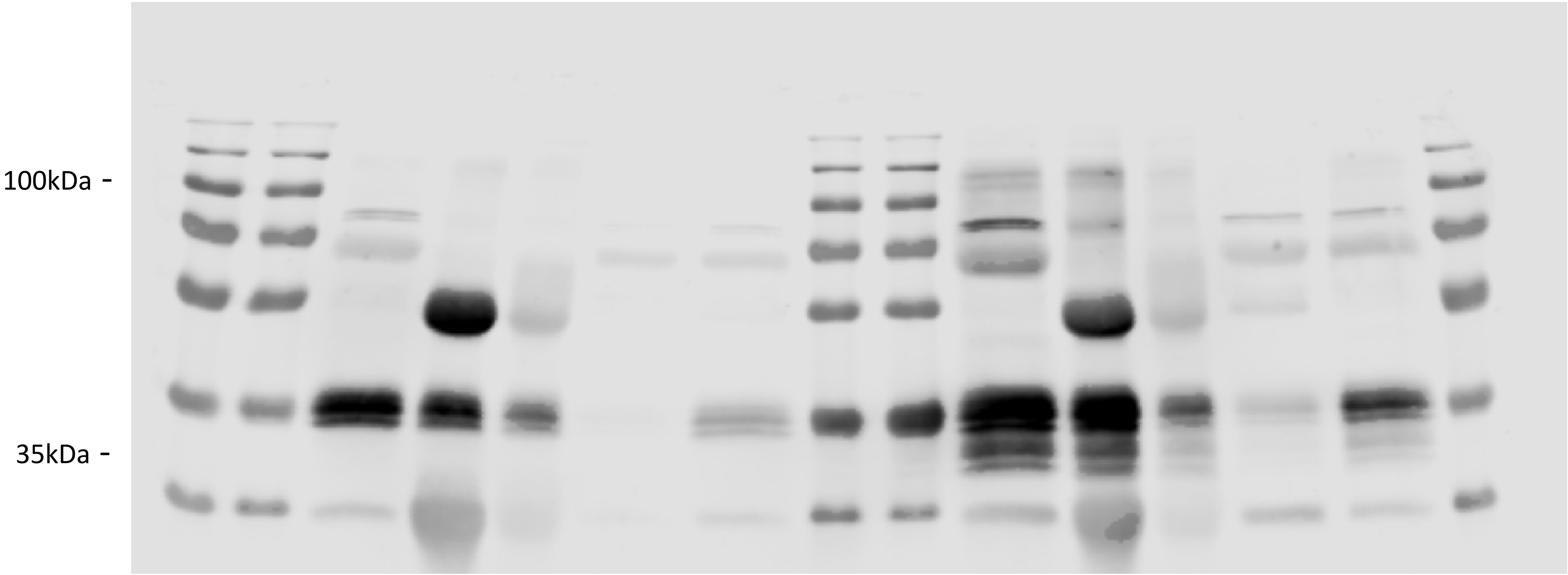

Lanes 3, 4, 6, 10, 11 and 13 used

Figure 2C. ApoE deglycosylation in human CSF

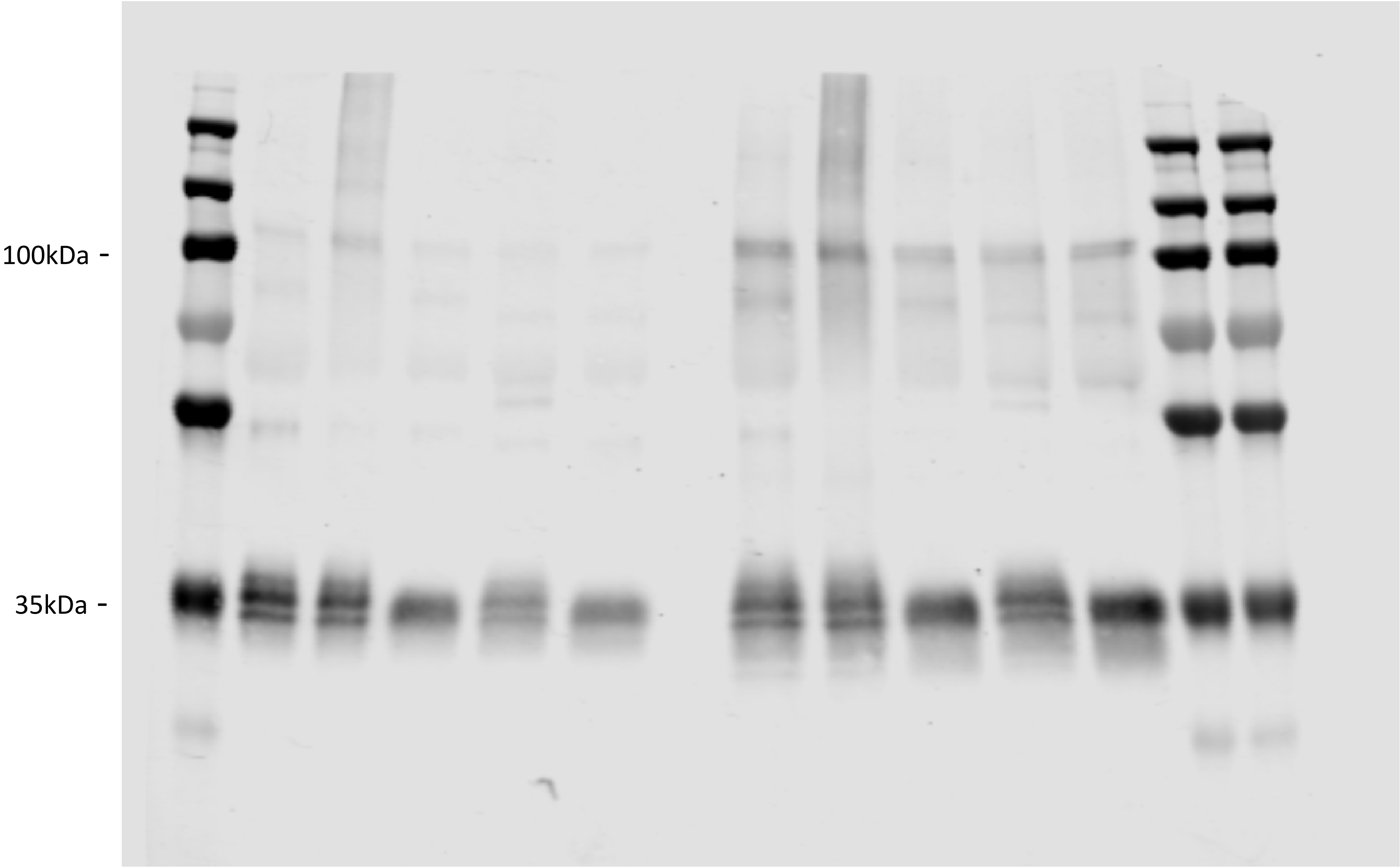

Figure 3A. ApoE under reducing and non-reducing conditions using standard anti-apoE antibody in human CSF

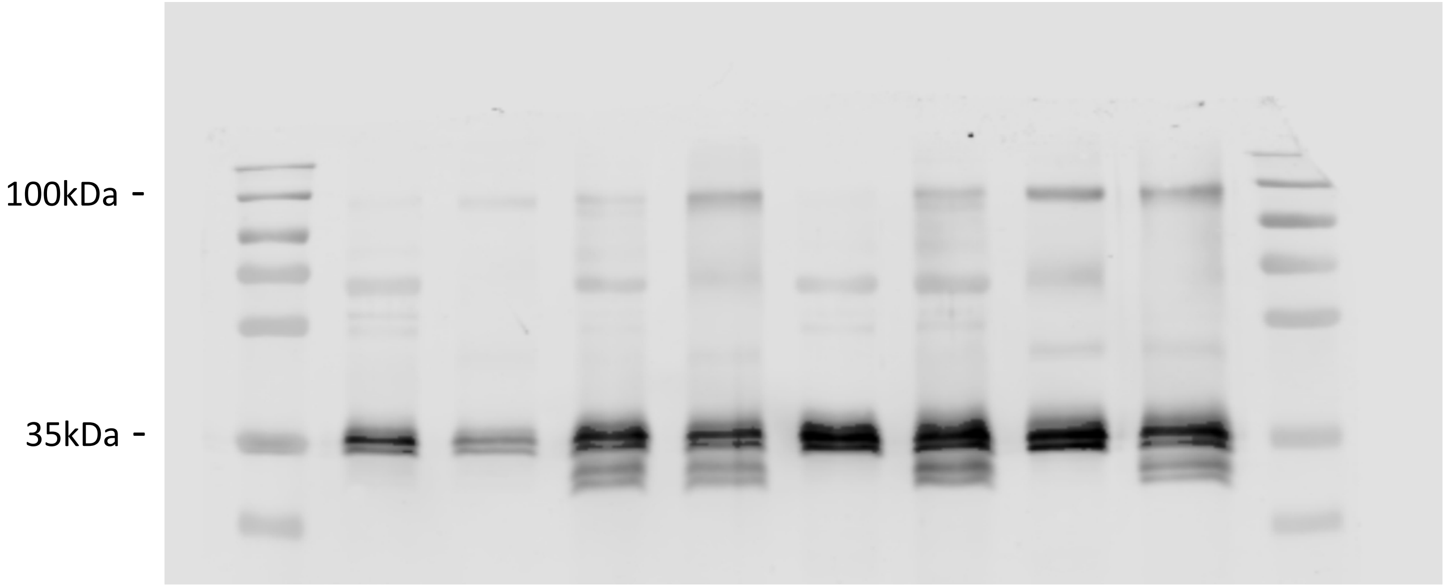

Lanes 5-8 used

Figure 3B. ApoE under reducing and non-reducing conditions using anti-apoE4 antibody in human CSF

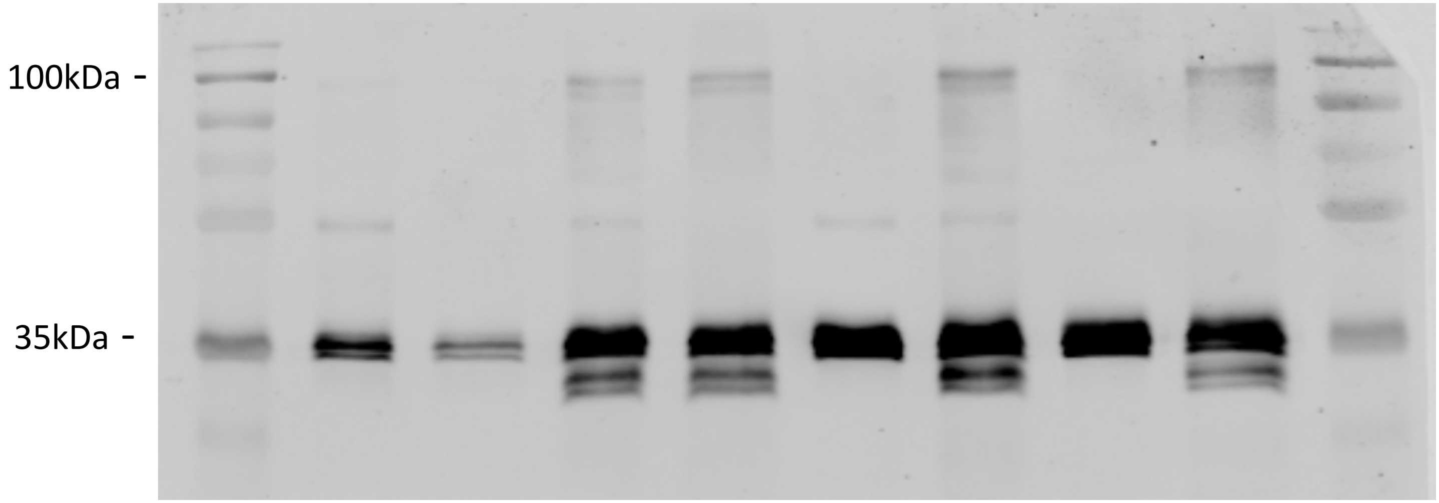

Lanes 5-8 used

Figure 3C. ApoE under native conditions in human CSF

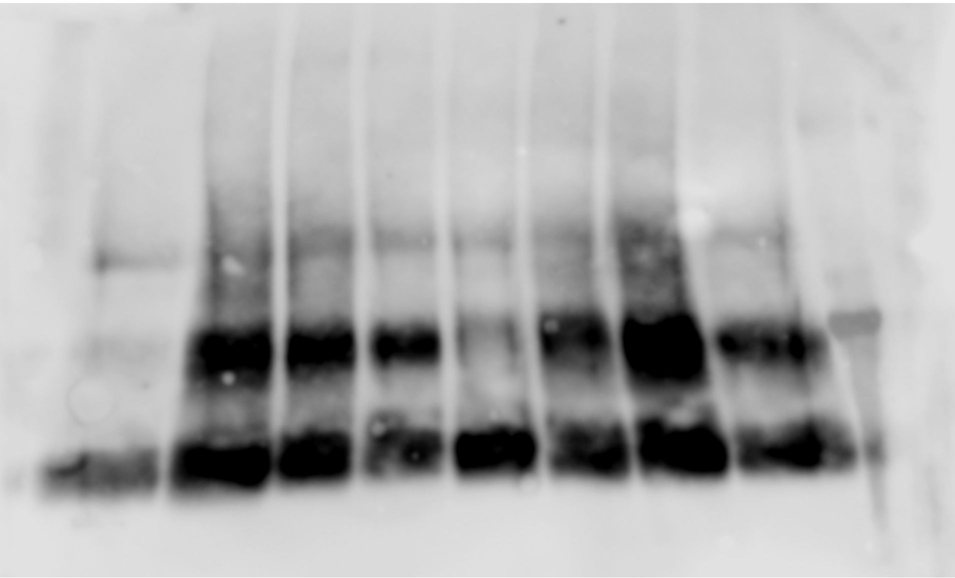

Lanes 3, 4 and 5 used

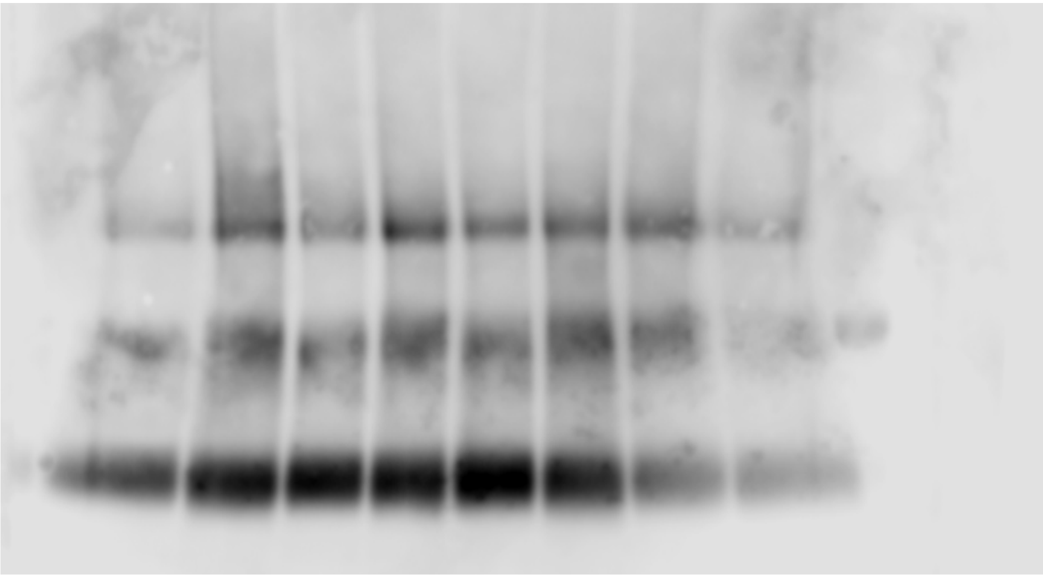

Lanes 5 and 6 used

Figure 4A. Representative blot of apoE in human CSF from the Gothenburg cohort

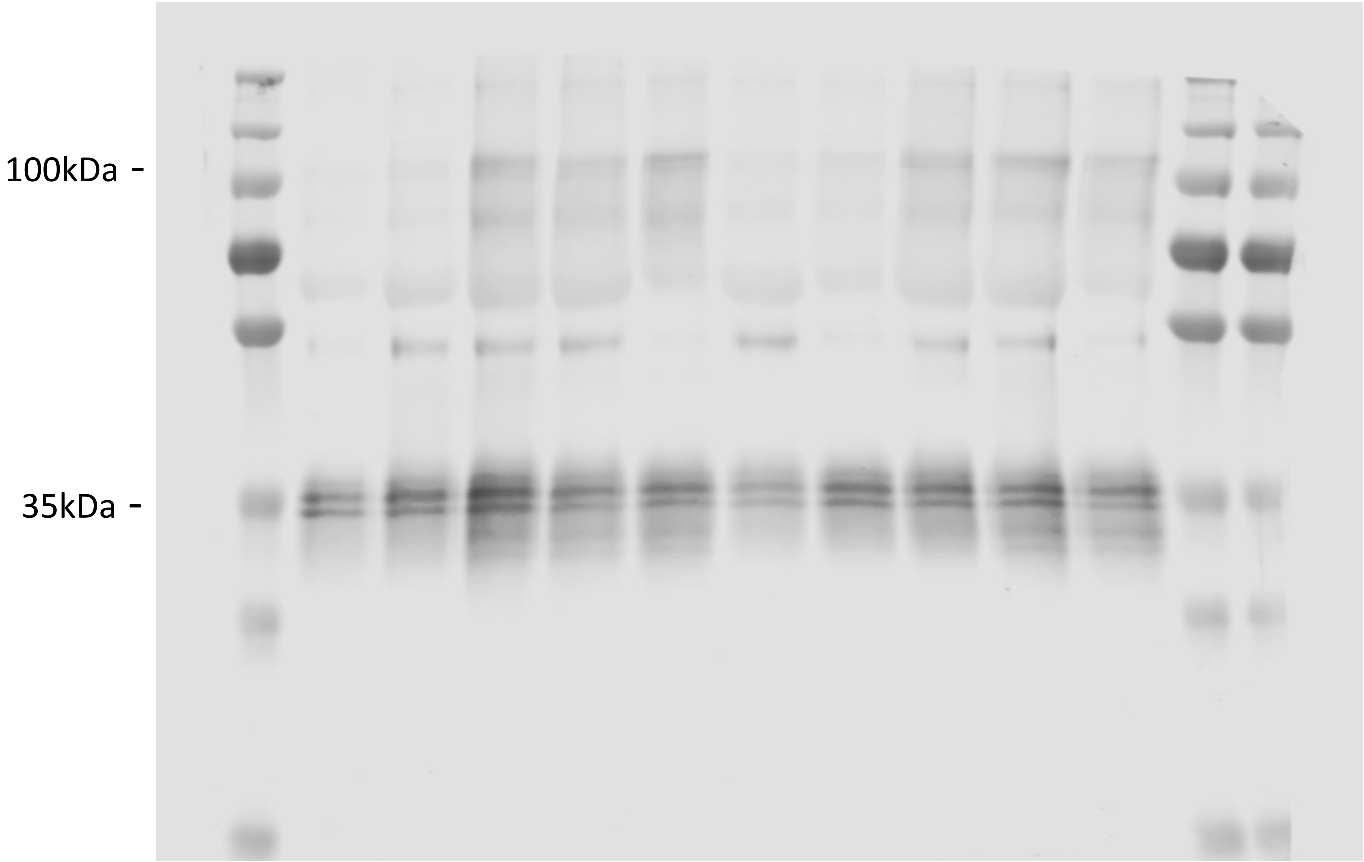

Lanes 2, 3, 4, 5 and 6 used

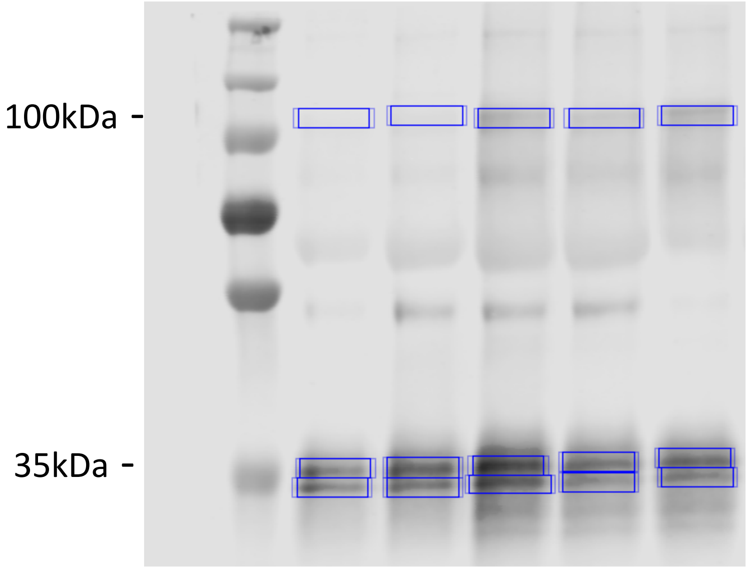

Figure 5A. Representative blot of apoE in human CSF from the Barcelona cohort

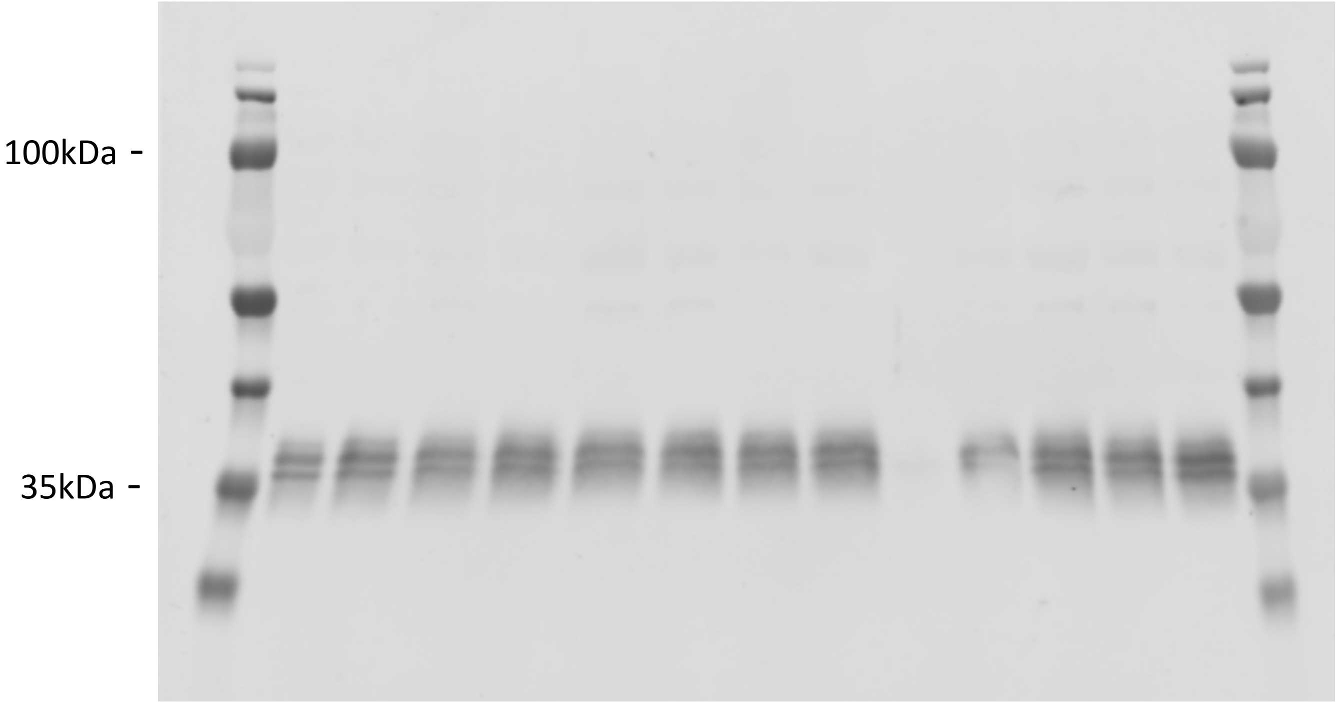

Lanes 3, 4, 12, 13 and 14 used
